# Effects of Compounds Isolated from *Lindera erythrocarpa* on Anti-Inflammatory and Anti-Neuroinflammatory Action in BV2 Microglia and RAW264.7 Macrophage

**DOI:** 10.3390/ijms23137122

**Published:** 2022-06-27

**Authors:** Chi-Su Yoon, Hwan Lee, Zhiming Liu, Hyeong-Kyu Lee, Dong-Sung Lee

**Affiliations:** 1 Natural Medicine Research Center, Korea Research Institute of Bioscience & Biotechnology (KRIBB), Cheongju-si 28116, Korea; ycs1991@naver.com (C.-S.Y.); brightjem6178@gmail.com (H.-K.L.); 2 Department of Chemistry, University of Florida, Gainesville, FL 32611, USA; 3 College of Pharmacy, Chosun University, Dong-gu, Gwangju 61452, Korea; ghksdldi123@hanmail.net (H.L.); lzmqust@126.com (Z.L.)

**Keywords:** *Lindera erythrocarpa*, bi-linderone, anti-inflammation, anti-neuroinflammation

## Abstract

*Lindera erythrocarpa* contains various constituents such as cyclopentenedione-, flavonoid-, and chalcone-type components. In this study, a novel bi-linderone derivative and 17 known compounds were isolated from the leaves of *L. erythrocarpa* by using various chromatographic methods. The structures of the components were determined from nuclear magnetic resonance and mass spectrometry data. All isolated compounds were tested for anti-inflammatory and anti-neuroinflammatory activities in lipopolysaccharide (LPS)-induced BV2 and RAW264.7 cells. Some of these compounds showed anti-inflammatory effects by inhibiting the nitric oxide (NO) produced by LPS. In particular, linderaspirone A (**16**), bi-linderone (**17**) and novel compound demethoxy-bi-linderone (**18**) showed significant inhibitory effects on the production of prostaglandin E2 (PGE_2_), tumor necrosis factor-α, and interleukin-6. The three compounds also inhibited the expression of inducible NO synthase (iNOS) and cyclooxygenase-2 (COX-2), which are pro-inflammatory proteins, and the activation of nuclear factor κB (NF-κB). Therefore, linderaspirone A (**16**), bi-linderone (**17**), and demethoxy-bi-linderone (**18**) isolated from the leaves of *L. erythrocarpa* have therapeutic potential in neuroinflammatory diseases.

## 1. Introduction

Neurodegenerative diseases are among the most common age-related diseases, affecting more than 50 million people worldwide [1]. Neurodegenerative diseases include Alzheimer’s disease (AD), Parkinson’s disease (PD), and Huntington’s disease (HD) [2]. Many studies have focused on developing treatments for neurodegenerative diseases, but these have not yet been developed. However, with the efforts of many researchers, neurodegenerative diseases have been found to begin with neuroinflammation [3,4,5]. Microglia and astrocytosis (gliosis) are involved in the regulation of neuroinflammation, and when they are activated during the induction of neurodegeneration, various inflammatory substances are secreted to induce neuroinflammation [6,7]. Thus, microglial activation and gliosis play a central role in neuroinflammation and the consequent neurodegeneration. Therefore, there is a need for more research on therapeutic agents for neurodegenerative diseases, with the signaling pathway of neuroinflammation as the target, and it is necessary to discover therapeutic agents that suppress neuroinflammation, the cause of the disease.

Microglia, which are the macrophages of the central nervous system, can activate and trigger innate immune responses by sensing exogenous neurotoxic substances and pro-inflammatory stimuli [8,9]. However, overactivation of microglia owing to various causes results in neuroinflammatory responses, nerve damage, and cognitive dysfunction [10]. Specifically, inflammation is mediated through the production of proinflammatory mediators including prostaglandin E2 (PGE_2_), tumor necrosis factor-α (TNF-α), interleukin-6 (IL-6), IL-1β, and nitric oxide (NO) [11]. Therefore, modulation of neuroinflammatory substances that can affect microglial activation represents a potential strategy for the alleviation of neurodegenerative diseases.

*Lindera erythrocarpa* is a plant belonging to the Lauraceae family and is widely distributed in Korea, Japan, and China [12]. The characteristic feature of *L. erythrocarpa* is that it blooms during April and May and bears fruits in September. Dried fruits have a distinctive aroma and bitter taste and are used in Japan as a digestive and pain reliever for neuralgia [13]. It has antifungal, digestive, and antibacterial properties and has been used as an herbal medicine for a long time. According to the findings of studies conducted thus far, various activities such as antioxidant, anti-inflammatory, anticancer, and antifungal activity of *L. erythrocarpa* have been reported. [14,15,16,17,18]. *L. erythrocarpa* contains various components such as linderone, lucidone, kanakuziol, camphene, and limonene [18,19,20]. However, among the compounds isolated from *L. erythrocarpa*, none of the studies have shown the inhibitory effects in neuroinflammation in microglia.

The purpose of this study was to identify bioactive and novel compounds in *L. erythrocarpa* leaves that inhibit neuroinflammation, a cause of neurodegenerative diseases. Therefore, using BV2 microglia and RAW264.7 macrophages, we attempted to isolate a novel component with an anti-inflammatory effect from the components present in the methanol extract of *L. erythrocarpa* leaves.

## 2. Results

### 2.1. Structural Elucidation of Isolated Compounds

**Figure 1:**
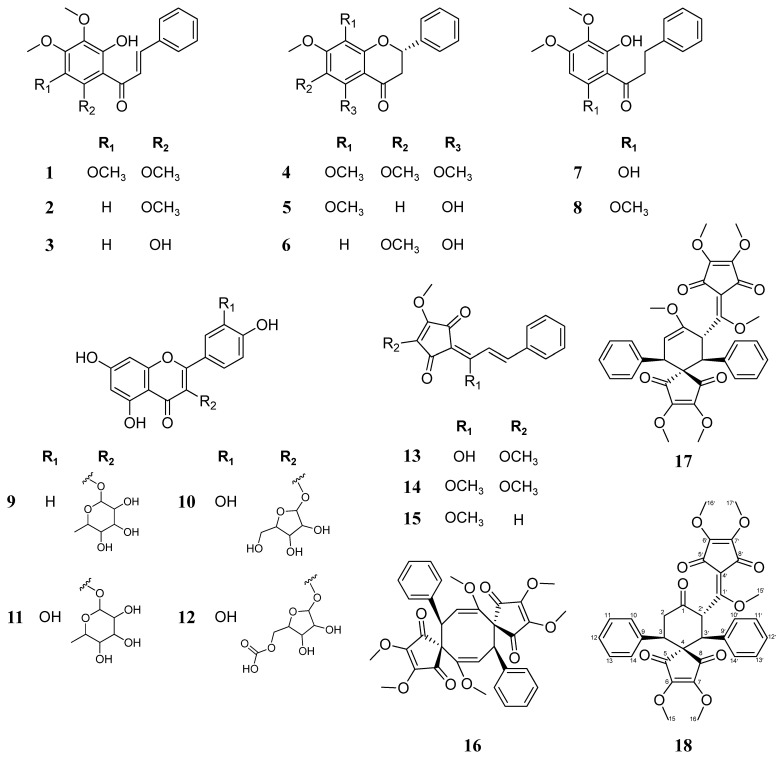
Structure of the compounds isolated from *L. erythrocarpa* leaves.

In this study, 18 compounds (Figure 1) were isolated from the leaves of *L. erythrocarpa* using several chromatographic methods. The chemical structures of the isolated compounds were determined from nuclear magnetic resonance (NMR) and mass spectrometry (*MS*) data. A novel bi-linderone derivative (**18**) and 17 known metabolites classified as chalcone, flavonoid, cyclopentenedione, and dimer of linderone were identified.

Demethoxy-bi-linderone (**18**) was isolated as white amorphous oil, and is based on the analysis of ^1^H- and ^13^C-NMR data (Table 1, Figures S18–S22) along with HR-ESI-MS data (*m*/*z* 587.1921 [M+H]^+^, calculated for C_33_H_31_O_10_^+^, 587.1912; 609.1707 [M+Na]^+^, calculated for C_33_H_30_O_10_Na^+^, 609.1731, Figure S23), its molecular formula was determined to be C_33_H_30_O_10_ with 19 unsaturation. The ^1^H-NMR data suggested the presence of two monosubstituted benzene rings at δ_H_ 7.03–7.24 (10H, m); five methoxyl groups at δ_H_ 3.62, 3.84, 3.89, 4.08, and 4.15; two groups of intercoupling protons at δ_H_ 2.66 (1H, dd, *J* = 14.0, 4.0 Hz) and 3.65 (1H, dd, *J* = 14.0, 4.0 Hz), as well as δ_H_ 6.53 (1H, d, *J* = 13.0 Hz) and 4.02 (1H, d, *J* = 13.0 Hz). The ^13^C-NMR and DEPT NMR spectra showed a total of 33 carbon signals and indicated the presence of five carbonyl groups at δ_C_ 183.6, 187.4, 195.0, 196.1, and 205.0; two monosubstituted phenyls at δ_C_ 137.4, 136.1 and 128.2–128.7; six olefinic carbons at δ_C_ 169.3, 153.4, 153.1, 148.3, 146.8, and 109.8; five methoxyls at δ_C_ 65.8, 60.1, 59.8, 59.61, and 59.56; a quaternary carbon at δ_C_ 58.8; three methine carbons at δ_C_ 54.5, 49.2, and 47.2; and a methylene carbon at δ_C_ 43.8. The NMR and mass data showed a structure similar to that of the previous bi-linderone type; particularly, **18** had a structure similar to that of demethoxy-epi-bi-linderone [21], but NMR chemical shifts were slightly different. The difference between **18** and demethoxy-epi-bi-linderone was similar to that between epi-bi-linderone and bi-linderone, which have different chiral centers in position 3 (Table S1) [22]. Therefore, we elucidated the structure of demethoxy-bi-linderone.

In addition, 17 known compounds were isolated in this study, and based on a comparison of mass and NMR data between the present study and previous literature, their structures were confirmed as kanakugiol (**1**) [23], 2′-hydroxy-3′,4′,6′-trimethoxychalcone (**2**) [24], pashanone (**3**) [25], kanakugin (**4**) [26], 5-hydroxy-7,8-dimethoxyflavanone (**5**) [27], onysilin (**6**) [27], dihydropashanone (**7**) [28], 2-hydroxy-3′,4′,6′-trimethoxydihydrochalcone (**8**) [29], avicularin (**9**) [30], afzelin (**10**) [30], avicularin-acetate (**11**) [31], quercitrin (**12**) [30], linderone (**13**) [32], methyllinderone (**14**) [32], methyllucidone (**15**) [32], linderaspirone A (**16**) [22], and bi-linderone (**17**) [22] (Figures S1–S17).

### 2.2. Inhibitory Effect of Nitrite Production of 18 Natural Compounds Isolated from L. erythrocarpa

**Figure 2:**
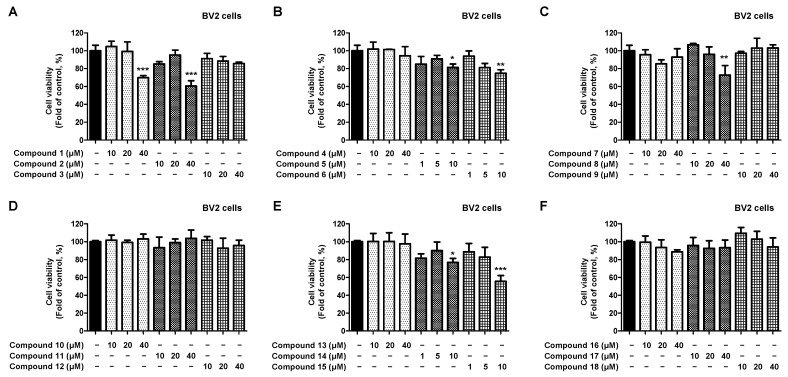
Cytotoxicity of the 18 natural compounds isolated from *L. erythrocarpa* leaves assayed in BV2 microglia cells (**A**–**F**). Cytotoxicity was assessed after treatment with the compounds at concentrations of 1–40 μM for 48 h. Data are presented as the mean ± standard deviation values of three independent experiments. * *p* < 0.05, ** *p* < 0.01, *** *p* < 0.001 vs. control.

To identify compounds that have an inhibitory effect on neuroinflammation and are present in *L. erythrocarpa* leaves, we investigated the anti-inflammatory effects of the 18 isolated compounds. First, toxicity was assessed to establish treatment concentrations for compounds isolated from BV2 microglia (Figure 2A–F). Compounds **3**, **4**, **7**, **9**–**13**, and **16**–**18** did not show toxicity even at a concentration of 40 μM, whereas compounds **1**, **2**, and **8** showed toxicity at a concentration of 40 μM. Compounds **5**, **6**, **14**, and **15** showed toxicity at 10 μM; therefore, the cell treatment concentration was set at 5 μM, which did not show toxicity.

**Figure 3:**
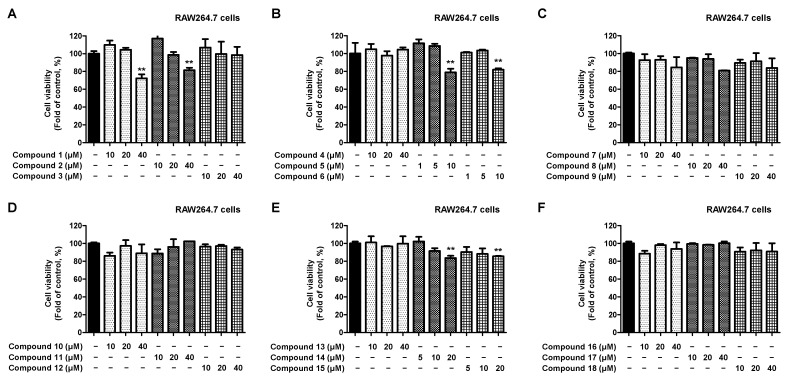
Cytotoxicity of the 18 natural compounds isolated from *L. erythrocarpa* leaves assayed in RAWA264.7 macrophage cells (**A**–**F**). Cytotoxicity was assessed after treatment with the compounds at concentrations of 1–40 μM for 48 h. Data are presented as the mean ± standard deviation values of three independent experiments. ** *p* < 0.01 vs. control.

Next, toxicity was assessed to establish treatment concentrations for compounds isolated from RAW264.7 macrophages (Figure 3A–F). Compounds **3**, **4**, **7**–**13**, and **16**–**18** did not show toxicity even at a concentration of 40 μM, whereas compounds **1** and **2** showed toxicity at a concentration of 40 μM. In addition, compounds **14** and **15** were toxic at a concentration of 20 μM, whereas compounds **5** and **6** were toxic at 10 μM. Based on these results, non-toxic treatment concentrations were established for all compounds.

**Figure 4:**
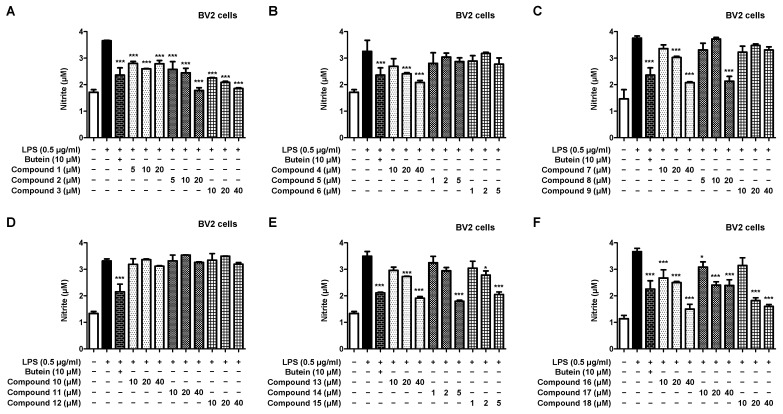
Inhibitory effect of the 18 natural compounds on nitrite production in BV2 microglia cells (**A**–**F**). The inhibitory effect on nitrite production in BV2 cells was induced for 18 h by pretreatment with the compound at a concentration of 1–40 μM followed by treatment with lipopolysaccharide (LPS). Data are expressed as the mean ± standard deviation value of three independent experiments. * *p* < 0.05, *** *p* < 0.001 vs. LPS.

We investigated the inhibitory effect of all compounds on nitrite production in lipopolysaccharide (LPS)-induced BV2 microglia under varied treatment concentration settings based on toxicity assessment (Figure 4A–F). The results confirmed that compounds **1**–**4**, **7**, **8**, and **13**–**18** inhibited nitrite production. In particular, compounds **9**–**12** showed similar flavonoid glycoside structures, and it was confirmed that none of them exhibited an inhibitory effect on nitrite production. In addition, it was confirmed that the novel compound, compound **18**, had a skeleton similar to those of compounds **16** and **17**, which were dimer of the linderone types, and had a similar inhibitory effect on nitrite production.

**Figure 5:**
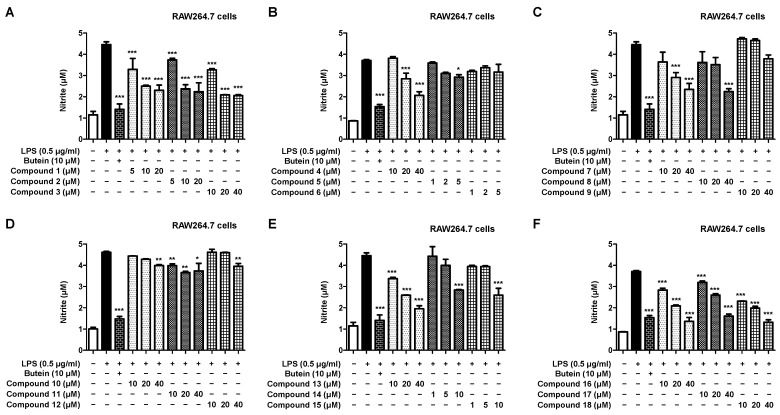
Inhibitory effect of the 18 natural compounds on nitrite production in RAW264.7 macrophage cells (**A**–**F**). The inhibitory effect on nitrite production in RAW264.7 cells was induced for 18 h by pretreatment with the compounds at a concentration of 1–40 μM followed by treatment with LPS. Data are expressed as the mean ± standard deviation value of three independent experiments. * *p* < 0.05, ** *p* < 0.01, *** *p* < 0.001 vs. LPS.

Next, we investigated the inhibitory effect of the native compounds on nitrite production in LPS-induced RAW264.7 macrophages (Figure 5A–F). It was confirmed that all compounds except compounds **6** and **9** had an inhibitory effect on nitrite production. In particular, it was confirmed that the novel compound **18** and the known compounds **16** and **17** had a nitrite production inhibitory effect that is, respectively, similar to or superior over the positive control at a concentration of 40 μM.

### 2.3. Inhibitory Effect of Compounds ***16**–**18*** on iNOS and COX-2 Expression

**Figure 6:**
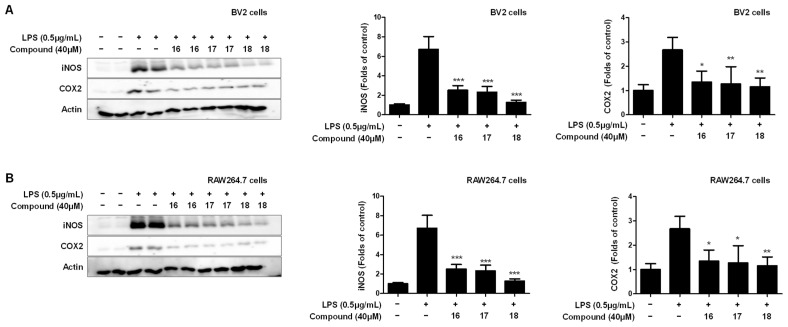
Effect of compounds **16**–**18** on protein expression levels of nitric oxide synthase (iNOS) and cyclooxygenase-2 (COX-2) in LPS-induced BV2 microglia (**A**) and RAW264.7 macrophages (**B**). The inhibitory effect on the expression of the pro-inflammatory proteins iNOS and COX-2 was induced in cells for 18 h by LPS treatment after pretreatment with 40 μM of compounds **16**–**18**. Representative blots of three independent experiments, obtained by Western blotting analysis, are shown. Data are expressed as the mean ± standard deviation value of three independent experiments. * *p* < 0.05, ** *p* < 0.01, *** *p* < 0.001 vs. LPS.

In this study, the inhibitory effect of the 18 natural compounds isolated from *L. erythrocarpa* leaves on nitrite production was confirmed using BV2 and RAW264.7 cells. Among them, additional activity evaluation was performed for compounds **16**–**18**, which showed the best inhibitory effect on nitrite production. To investigate the additional anti-inflammatory effect of the compound, the inhibitory effect of the pro-inflammatory proteins inducible NO synthase (iNOS) and cyclooxygenase-2 (COX-2) expression in LPS-induced BV2 and RAW264.7 cells was investigated (Figure 6). The compounds **16**–**18** inhibited the expression of both iNOS and COX-2, which are pro-inflammatory proteins. In particular, compound **18** exhibited the strongest inhibitory effect on the expression of the pro-inflammatory proteins.

### 2.4. Inhibitory Effect of Compounds ***16**–**18*** on PGE_2_, TNF-α, and IL-6 Production

**Figure 7:**
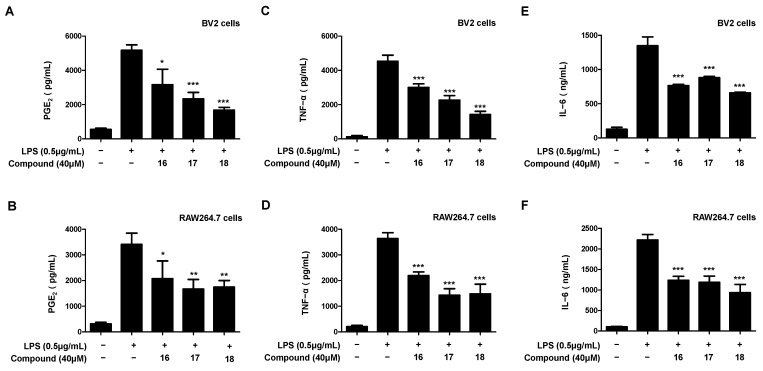
Inhibitory effect of compounds **16**–**18** on the inflammatory mediators PGE_2_, TNF-α, and IL-6 in LPS-induced BV2 microglia (**A**,**C**,**E**) and RAW264.7 macrophages (**B**,**D**,**F**). The inhibitory effect on PGE_2_, TNF-α, and IL-6 production in BV2 and RAW264.7 cells was induced for 18 h by treatment with LPS after pretreatment with 40 μM of the compounds. Data are presented as the mean ± standard deviation values of three independent experiments. * *p* < 0.05, ** *p* < 0.01, *** *p* < 0.001 vs. LPS.

First, we confirmed the effects on the production of inflammatory mediators and cytokine when each compound of 16, 17, and 18 was treated to the BV2 and RAW264.7 cells without stimulus. As a result, compounds **16**–**18** had no effect on nitrite, PGE_2_, TNF-α and IL-6 production when treated alone (Figure S24). Through Western blotting, we confirmed that compounds **16**–**18** isolated from *L. erythrocarpa* leaves inhibited the expression of pro-inflammatory proteins. Therefore, we investigated whether these compounds modulated the release of PGE_2_, TNF-α, and IL-6, which are substances that modulate cellular inflammation associated with the expression of pro-inflammatory proteins. First, the inhibitory effect on the production of PGE_2_, an inflammatory mediator synthesized by the expression of the COX-2 protein in LPS-induced BV2 and RAW264.7 cells, was confirmed (Figure 7A,B). All three compounds inhibited the production of PGE_2_, and compound **18** exhibited the best inhibitory effect on the production of PGE_2_. Next, we investigated whether it modulates the release of the inflammatory cytokines TNF-α and IL-6 from cells in response to inflammation (Figure 7C–F). All three compounds inhibited the release of TNF-α and IL-6 from cells, and compound **18** had the best inhibitory effect on the release of inflammatory cytokines in both BV2 and RAW264.7 cells.

### 2.5. Inhibitory Effect of Compounds ***16**–**18*** on NF-κB Activation

We found that compounds **16**–**18** isolated from *L. erythrocarpa* leaves modulated inflammatory mediators. Therefore, it was necessary to investigate whether compounds **16**–**18** were involved in the activation of nuclear factor κB (NF-κB), which regulates iNOS and COX-2 expression. Therefore, it was investigated whether the compounds regulate the activation of NF-κB in LPS-induced BV2 and RAW264.7 cells (Figure 7). All three compounds inhibited the activation of NF-κB, and it was found that compound **18** had the strongest inhibitory effect on the activation of NF-κB.

## 3. Discussion

*L. erythrocarpa* has long been used in traditional medicine and has been extensively studied for its phytochemical components and biological activity. To date, several research papers have shown that *L. erythrocarpa* contains various components such as cyclopentedione- and flavonoid-type of compounds; their various activities have been studied as well. We attempted to isolate the bioactive and novel natural compound from *L. erythrocarpa* leaves, and as a result, one novel and 17 known compounds were isolated. Most compounds have already been reported as the components of *L. erythrocarpa*, but their bioactive for anti-neuroinflammatory effects have not yet been investigated. Therefore, we first attempted to compare the anti-inflammatory activities of the compounds isolated using LPS-induced BV2 microglia and RAW264.7 macrophages. 

Macrophages and microglia are activated when harmful factors are present and play an important role in maintaining homeostasis by eliminating side effects in the body. However, when cells are overactivated, they secrete pro-inflammatory mediators and inflammatory cytokines [33]. The secretion of inflammatory mediators causes cell damage and, if left unattended, brain damage, leading to degenerative brain disease [34,35]. Therefore, a therapeutic agent to prevent degenerative brain disease should exhibit the effect of suppressing the secretion of inflammatory mediators. LPS is a major component of the outer cell membrane of Gram-negative bacteria. It is a substance in which lipids and polysaccharides are covalently bound to each other. As a pathogenic factor induced by an inflammatory response, it is widely used in anti-inflammatory studies, including microglia [36]. When macrophages and microglia are overactivated by LPS, inflammatory substances such as nitrite and PGE_2_ are secreted. Therefore, confirming the nitrite production inhibitory effect is a measure for confirming the anti-inflammatory effect, and the inhibitory effect of the isolated compound on nitrite production was compared (Figure 4 and Figure 5). Compounds **1**–**4**, **7**, **8**, and **13**–**18** inhibited nitrite production in LPS-induced BV2 cells. In addition, compounds **1**–**5**, **7**, **8**, and **10**–**18** inhibited nitrite production in LPS-induced RAW264.7 cells. Compounds **6** and **9** did not inhibit nitrite production in either BV2 or RAW264.7 cells. In particular, it was confirmed that the novel compound **18** and known compounds **16** and **17** had a nitrite production inhibitory effect that is similar to or superior over the positive control at a concentration of 40 μM in both macrophages and microglia.

The compounds isolated in this study were classified into five groups according to their structures. This is because similar activity was confirmed depending on the structure type while confirming the nitrite production inhibitory effect. Compounds **1**–**3**, **7**, and **8** were identified as chalcone-type compounds. Chalcone has an α,β-unsaturated ketone with various pharmacological activities, including antioxidant, anti-inflammatory, anticancer, antiviral, and immunosuppressive [37]. Kanakugiol (**1**) was isolated from *L. erythrocarpa* [17], and the activity of **1** has been reported as free radical scavenging, antifungal, and anticancer [17,18,38]. 2′-hydroxy-3′,4′,6′-trimethoxychalcone (**2**) was previously isolated from Polygonaceae, Annonaceae, Piperaceae, and Rosaceae, and this study is the first to report isolation from *L. erythrocarpa* [29,39]. Compound **2** is known to have cytotoxic effects, and most compounds with a chalcone structure are similar to that of various active ingredients, including antioxidant and anti-inflammatory effects [39]. Pashanone (**3**) has been reported to be isolated from Polygonaceae [39]. Its known activity was similar with compound **2** [39]. There are only a few reports on Dihydropashanone (**7**) and 2′-hydroxy-3′,4′,6′-trimethoxydihydrochalcone (**8**) and the activity of these compounds. However, it was expected to show general activity of the chalcone type, and it was confirmed that nitrite was significantly inhibited in BV2 microglia and RAW264.7 macrophages. In this study, we confirmed that compounds **1**–**3**, **7**, and **8** have similar inhibitory effects on nitrite production in BV2 microglia and RAW264.7 macrophages. However, the anti-inflammatory effects of chalcone-type compounds are well known; thus, no further studies have been conducted [37].

Compounds **4**–**6** were identified as flavanone-type compounds in this study. Flavanones have a stereogenic center (chiral center) at the C-2 position and various pharmacological activities, including antioxidant, cytotoxic, antibacterial, and quinone reductase activities [40]. Kanakugin (**4**) was isolated from *L. erythrocarpa*, and its various activities including skin protection have been reported [41]. 5-hydroxyl-7,8-dimethoxyflavanone (**5**) was discovered as a novel compound in *Fistigma cupreonitens* and has been reported to reverse drug resistance in cancer [42]. Onysilin (**6**) has been known as the component in the bark of *L. oxyphylla* and the same genus. In this study, flavanone-type compounds such as compounds **4**–**6** did not effectively inhibit nitrite production compared with other types of compounds. Therefore, we did not conduct follow-up studies.

Compounds **9**–**12** were considered as the flavonol glycosides group. Although flavonols are widely known for their antioxidant, antihyperlipidemic, and anticancer effects, especially, the antioxidant effect of flavonol glycosides is reported to be weaker than that of flavonol aglycones [43,44,45]. The flavonol glycosides isolated in this study had weaker effects than other types of flavonoid compounds. These results showed the same aspect as the above-mentioned antioxidant effect between flavonol glycosides and aglycones.

Compounds **13**–**15** are identified as cyclopentadione, and these are known as major components of *L. erythrocarpa* [16]. These compounds were previously reported to have anti-inflammatory effects on macrophages [16]. Therefore, we did not investigate the anti-inflammatory effects of these three compounds.

Compounds **16** and **17**, as well as the epimer of **18**, were previously isolated from *Lindera aggregata* [21,46,47]. Compounds **16** and **17** were reported as possible to synthesize from methyllinderone (**14**) reacted by UV. Furthermore, these two have also been reported to exhibit significant activity against glucosamine-induced insulin resistance in HepG2 cells [22]. However, studies on the anti-neuroinflammatory effects related to degenerative brain diseases have not yet been conducted. Both compounds showed a structure similar to that of the novel compound **18**. In addition, among the various structural types isolated from *L. erythrocarpa* leaves, it was found that compounds 16, 17 and 18 of the dimer of linderone type showed the best anti-inflammatory effect. Therefore, we selected compounds **16**–**18** for further investigation of their anti-inflammatory activity.

In the process of macrophage inflammatory response, inflammatory mediators are generated by the expression of pro-inflammatory proteins. The most important proteins that generate inflammatory mediators are iNOS and COX-2. Expression of iNOS is induced by various cytokines released by inflammatory and immune responses, tissue damage, and oxidative stress at the gene transcription stage to generate large amounts of NO [48]. COX-2 plays a role in promoting cell proliferation by inducing the production of large amounts of prostaglandins in the inflammatory process and increasing the inflammatory response by suppressing immunity [49]. Therefore, we performed additional mechanistic studies of compounds **16**–**18** for understanding the regulation of iNOS and COX-2, which are important factors regulating the inflammatory response (Figure 6). All compounds inhibited the expression of the pro-inflammatory proteins iNOS and COX-2 in LPS-induced BV2 and RAW264.7 cells. Next, the inflammatory cytokine and inflammatory mediator PGE_2_, which induces the expression of pro-inflammatory proteins, were investigated (Figure 7). As expected, all compounds inhibited the inflammatory cytokines TNF-α and IL-6, including PGE_2_. By investigating the mechanisms of pro-inflammatory proteins and inflammatory cytokines, compound **18** was found to have the best anti-neuritis and anti-inflammatory effects.

**Figure 8:**
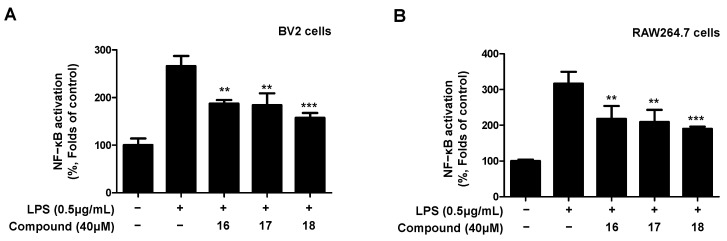
Inhibitory effect of compounds **16**–**18** on nuclear NF-κB activation in LPS-induced BV2 microglia (**A**) and RAW264.7 macrophages (**B**). The inhibitory effect on NF-κB activation in BV2 and RAW264.7 cells was induced for 18 h by treatment with LPS after pretreatment with 40 μM of the compounds. Data are presented as the mean ± standard deviation values of three independent experiments. ** *p* < 0.01, *** *p* < 0.001 vs. LPS.

We further investigated the anti-inflammatory mechanisms of the three compounds, including the novel ones, by measuring NF-κB binding activity (Figure 8). NF-κB belongs to the rel family and consists of the homo- and heterodimeric forms. The most widely studied form of NF-κB is a heterodimer of the p50 and p65 subunits, and it is a potent activator of gene transcription. In cells without activity, NF-κB is mainly present in the cytoplasm in an inactive state; however, when activated, the NF-κB heterodimer, which forms a complex of p65 and p50, enters the nucleus and is activated [50]. Activated NF-κB induces pro-inflammatory mediators and cytokines including NO, PGE_2_, TNF-α, IL-6, iNOS, and COX-2. In particular, it is significantly associated with NF-κB activation in chronic inflammation-related diseases such as asthma, arteriosclerosis, acquired immunodeficiency syndrome, AD, and PD [51,52]. This finding is consistent with the target disease of this study, and we need to identify compounds that inhibit NF-κB activation. Compounds **16**–**18** were confirmed to have inhibitory effects on NF-κB binding activity. In addition, the degree of inhibition of each compound showed a tendency similar to that of pro-inflammatory proteins and cytokines. Therefore, our results are the first to report the anti-neuroinflammatory effects of compounds **16**–**18**, and one novel compound discovered in this study was confirmed to have potential as a therapeutic agent for neurodegenerative diseases. However, it is necessary to confirm the therapeutic effect of these compounds on neurodegenerative diseases using additional anti-neuroinflammatory mechanisms and in vivo models.

## 4. Materials and Methods 

### 4.1. Plant Materials

One- and two-dimensional NMR spectra were recorded in chloroform-*d*, and dimethyl sulfoxide (DMSO)-*d*_6_ using a JEOL JNM ECP-400 spectrometer (400 MHz for ^1^H and 100 MHz for ^13^C). Electrospray ionization mass spectrometry (ESIMS) data were obtained using a quadrupole time-of-flight mass spectrometer (Q-TOF) micro-liquid chromatography–mass spectrometry (LC-MS/MS) instrument (Waters, Milford, MA, USA) at the Korea Research Institute of Bioscience and Biotechnology (KRIBB), Ohchang, Korea. Solvents used for extraction and flash column chromatography (CC) were of reagent grade and were used without further purification. The solvents used for high-performance liquid chromatography (HPLC) were of analytical grade. Flash CC was performed using YMC octadecyl-functionalized silica gel (C18) and silica gel (Merck, Darmstadt, Germany). HPLC separations were performed on a prep-C18 column (21.2 × 250 mm; 5 μm particle size) with a flow rate of 10 mL/min, and a semiprep-C18 column (10 × 250 mm; 5 μm particle size) with a flow rate of 3 mL/min.

### 4.2. Plant Material and Extraction

The leaves of *Lindera erythrocarpa* Makino were collected in October 2018 from the back yard at Gongju National Museum, South Korea. The species identified by Dr. Hyung-Kyu Lee and the voucher specimen were deposited in the Korea Plant Extract Bank of KRIBB (KPM038-045). Dried and ground leaves (1.5 kg) were extracted in 10 L of methanol (MeOH) with sonication for 3 h and (repeated five times) to obtain 171 g of methanol extract (LE). The methanolic extracts were dissolved in a mixture of 90% methanol and 10% water (4 L) and sequentially partitioned with 10 L each of *n*-hexane, ethyl acetate (EA), and *n*-butanol. The LE hexane fraction (LEH) was subjected to silica gel CC and eluted with hexane:EA (20:1 to 1:10) and EA:MeOH (1:0 to 0:1) to yield 35 fractions, LEH-1 to -35. The subfractions LEH-11 to -13 were combined (LEH-A), subjected to C18 CC, and eluted with MeOH:water (7:3 to 1:0) to yield 13 fractions, LEH-A-1 to -13. The subfraction LEH-A-5 was subjected to silica gel CC and eluted with CHCl_3_:EA:MeOH (50:1:0.1 to 1:1:0.1) to give compound **1** (3.1 g) and three fractions, LEH-A-5-1 to -4. Subfraction LEH-A-5-4 was subjected to prep-HPLC and eluted with a gradient system of 60–100% acetonitrile (AcN) in water (0.1% formic acid) over 30 min to yield **4** (150 mg, R_T_ = 12.2 min). Subfractions LEH-17 and -18 were combined (LEH-B), dissolved in *n*-hexane, and partitioned with 60% MeOH in water to remove the chlorophyll components, and two fractions were obtained: LEH-B-1 and -Hex. The subfraction LEH-B-1 was subjected to C18 CC and eluted with MeOH:water (6:4 to 1:0) to yield eight fractions, LEH-B-1-1 to -8. The subfractions LEH-B-1-5 to -7 were subjected to prep-HPLC and eluted with a gradient system of 60–77% AcN in water (0.1% formic acid) over 27 min to obtain **7** (33 mg, R_T_ = 20.2 min), and the fraction that was combined with a similar UV pattern was obtained and named LEH-D. The subfraction LEH-19 was dissolved in hexane and partitioned with 70% MeOH in water to remove the chlorophyll components, and two fractions were obtained: LEH-19-1 and -Hex. The subfraction LEH-19-1 was subjected to C18 CC and eluted with MeOH:water (6:1 to 1:0) to yield seven fractions, LEH-19-1-1 to -7. The subfractions LEH-19-1-5 to -7 were combined (LEH-19-1-A), subjected to silica gel CC, and eluted with hexane:acetone (7:1 to 0:1) to give eight fractions, LEH-19-1-A-1 to -8. Subfraction LEH-19-1-A-7 was dissolved in CHCl_3_:MeOH (1:1) to obtain crystalized compound **3** (100 mg). The subfraction LEH-D was subjected to C18 CC and eluted with AcN:water (55:45 to 1:0) to yield seven fractions, LEH-D-1 to -7. The fraction LEH-D-3 determined structure by analysis of 1D- and 2D-NMR, but NMR data (Figure S5) showed that this fraction contains two compounds as the ratio (8:2), therefore, two compounds were purified by additional following HPLC method. Subfraction LEH-D-3 was subjected to prep-HPLC and eluted with a gradient system of 70–80% MeOH in water (0.1% formic acid) over 50 min to yield **5** (8.6 mg, R_T_ = 19.5) and **6** (1.9 mg, R_T_ = 21.3). The subfraction LEH-D-6 was subjected to silica gel CC and eluted with hexane:acetone (8:1) to yield **14** (400 mg). The subfractions LEH-21 to -24 were combined (LEH-C), subjected to C18 CC, and eluted with MeOH:water (7.5:2.5 to 1:0) to give nine fractions, LEH-C-1 to -9. The subfraction LEH-C-3 was dissolved in CHCl_3_:MeOH (8:2) to obtain crystallized compound **15** (980 mg). The subfraction LEH-C-4 was subjected to prep-HPLC and eluted with a gradient system of 62–80% AcN in water (0.1% formic acid) over 30 min to yield **2** (30 mg, R_T_ = 21.2) and **8** (4 mg, R_T_ = 22.1). The subfractions LEH-B-1-6-3, -B-1-7-3, -D-6-5-3, and D-7-4 were combined (LEH-E-1) because these fractions showed similar patterns in thin-layer chromatography (TLC) analysis, and they were subjected to silica gel CC and eluted with hexane:EA (2:1) to yield six fractions, LEH-E-1-1 to -6. Subfraction LEH-E-1-3 was subjected to prep-HPLC and eluted with a gradient system of 60–80% AcN in water (0.1% formic acid) over 35 min to yield **17** (10.2 mg, R_T_ = 26.4). The subfractions LEH-E-1-4 and -5 were combined (LEH-E-1-A), subjected to semiprep-HPLC, and eluted with a gradient system of 55–62% AcN in water (0.1% formic acid) over 40 min to yield **16** (14 mg, R_T_ = 28.6 min). The subfractions LEH-D-6-5-2 and D-7-3 were combined (LEH-E-2) because these fractions showed similar patterns in TLC analysis and were subjected to prep-HPLC and eluted with a gradient system of 63–67% AcN in water (0.1% formic acid) over 30 min to yield **18** (31 mg, R_T_ = 20.8). The LE EA fraction (LEE) was subjected to silica gel CC and eluted with hexane:EA (10:1 to 1:10) and EA:MeOH (1:0 to 0:1) to yield 24 fractions, LEE-1 to -24. The subfractions LEE-19 and -20 were combined (LEE-A), subjected to silica gel CC, and eluted with CHCl_3_:MeOH (15:1 to 1:1) to give 11 fractions, LEE-A-1 to -11. The subfraction LEE-A-5 was subjected to silica gel CC and eluted with hexane:EA:MeOH (1:5:0.3) to yield eight fractions, LEE-A-5-1 to -8. The subfractions LEE-A-5-3, LEE-A-6, and LEE-A-8 were subjected to pre-HPLC and eluted with the same gradient system of 25–55% AcN in water (0.1% formic acid) over 30 min to yield **11** (from LEE-A-5-3, 20 mg, R_T_ = 23.8), **9** (from LEE-A-6, 120 mg, R_T_ = 21.3), **10** (from LEE-A-6, 78 mg, R_T_ = 23.3), and **12** (from LEE-A-8, 116 mg, R_T_ = 20.9). The subfractions LEE-15 and -16 were combined (LEE-B), subjected to C18 CC, and eluted with MeOH:water (4:6 to 1:0) to yield 11 fractions, LEE-B-1 to -11. Subfraction LEE-B-10 was subjected to prep-HPLC and eluted with a gradient system of 50–100% AcN in water (0.1% formic acid) to yield **13** (10 mg, R_T_ = 23.3).

Kanakugiol (**1**): ^1^H NMR (500 MHz, CDCl_3_); δ 13.20 (s, OH-6′), 7.94 (1H, d, *J* = 15.6 Hz, H-α), 7.85 (1H, d, *J* = 15.6 Hz, H-β), 7.65 (2H, dd, *J* = 6.4, 2.8 Hz, H-2, -6), 7.43 (3H, m, H-3, -4, -5), 4.11 (3H, s, OCH_3_-2′), 3.90 (6H, s, OCH_3_-3′, -5′), 3.87 (3H, s, OCH_3_-4′). ^13^C NMR (125 MHz, CDCl_3_); δ 194.0 (C-β′), 155.3 (C-2′), 153.8 (C-4′), 151.2 (C-6′), 144.0 (C-β), 138.7 (C-5′), 137.4 (C-3′), 135.4 (C-1), 130.7 (C-4), 129.2 (C-3, -5), 128.7 (C-2, -6), 126.7 (C-α), 111.2 (C-1′), 62.4 (OCH_3_-2′), 61.8 (OCH_3_-5′), 61.6 (OCH_3_-3′), 61.3 (OCH_3_-4′) (Figure S1).

2′-Hydroxy-3′,4′,6′-trimethoxychalcone (**2**): ^1^H NMR (500 MHz, CDCl_3_); δ 13.93 (s, OH-6′), 7.89 (1H, d, *J* = 15.6 Hz, H-α), 7.80 (1H, d, *J* = 15.6 Hz, H-β), 7.61 (2H, dd, *J*= 7.3, 2.5 Hz, H-2, -6), 7.41 (3H, m, H-3, -4, -5), 6.02 (1H, s, H-3′), 3.97 (6H, s, OCH_3_-2′, -4′), 3.85 (3H, s, OCH_3_-5′). ^13^C NMR (125 MHz, CDCl_3_); δ 193.5 (C-β′), 159.6 (C-6′), 158.8 (C-4′), 158.7 (C-2′), 142.9 (C-β), 135.7 (C-1), 131.1 (C-5′), 130.4 (C-α), 129.1 (C-3, -5), 128.6 (C-2, -6), 127.7 (C-4), 107.1 (C-1′), 87.3 (C-3′), 61.0 (OCH_3_-5′), 56.3 (OCH_3_-2′), 56.2 (OCH_3_-4′) (Figure S2).

Pashanone (**3**): ^1^H NMR (500 MHz, DMSO-*d*_6_); δ 8.12 (1H, d, *J* = 15.7 Hz, H-α), 7.73 (1H, d, *J* = 15.7 Hz, H-β), 7.68 (2H, dd, *J* = 7.5, 1.7 Hz, H-2, -6), 7.45 (3H, m, H-3, -4, -5), 6.12 (1H, s, H-5′), 3.83 (3H, s, OCH_3_-3′), 3.65 (3H, s, OCH_3_-4′). ^13^C NMR (125 MHz, DMSO-*d*_6_); δ 192.6 (C-β′), 160.0 (C-4′), 159.2 (C-6′), 154.7 (C-2′), 142.1 (C-β), 135.0 (C-3′), 130.4 (C-4), 129.1 (C-2, -6), 128.8 (C-1), 128.3 (C-3, -5), 127.4 (C-α), 105.1 (C-1′), 91.6 (C-5′), 60.3 (OCH_3_-3′), 55.9 (OCH_3_-4′) (Figure S3).

Kanakugin (**4**): ^1^H NMR (500 MHz, CDCl_3_); δ 7.48 (2H, d, *J* = 7.3 Hz, H-2′, -6′), 7.43 (2H, t, *J* = 7.4 Hz, H-3′, -5′), 7.38 (1H, t, *J* = 7.2 Hz, H-4′), 5.45 (1H, dd, *J* = 12.9, 2.8 Hz, H-2), 4.07 (3H, s, OCH_3_-5), 3.91 (3H, s, OCH_3_-8), 3.86 (6H, s, OCH_3_-6, 7), 3.02 (1H, dd, *J* = 16.6, 13.0 Hz, H-3α), 2.87 (1H, dd, *J* = 12.9, 2.8 Hz, H-3β). ^13^C NMR (125 MHz, CDCl_3_); δ 189.9 (C-4), 153.6 (C-7), 152.6 (C-5), 150.3 (C-8a), 141.3 (C-8), 138.9 (C-6), 138.0 (C-1′), 129.0 (C-2′, -6′), 128.8 (C-4′), 126.1 (C-3′, -5′), 111.7 (C-4a), 79.5 (C-2), 61.9 (OCH_3_-5), 61.8 (OCH_3_-8), 61.7 (OCH_3_-6), 61.6 (OCH_3_-7), 46.1 (C-3) (Figure S4).

5-Hydroxyl-7,8-dimethoxyflavanone (**5**): ^1^H NMR (500 MHz, CDCl_3_); δ 11.98 (1H, s, OH-5), 7.38–7.47 (5H, m, H-2′-6′), 6.12 (1H, s, H-6), 5.48 (1H, dd, *J* = 12.1, 3.3 Hz, H-2), 3.90 (3H, s, OCH_3_-7), 3.79 (3H, s, OCH_3_-8), 3.09 (1H, dd, *J* = 17.2, 12.1 Hz, H-3α), 2.89 (1H, dd, *J* = 17.2, 3.3 Hz, H-3β). ^13^C NMR (125 MHz, CDCl_3_); δ 196.2 (C-4), 161.8 (C-7), 160.1 (C-5), 153.8 (C-8a), 138.7 (C-1′), 130.0 (C-8), 129.1 (C-4′), 129.0 (C-3′, -5′), 128.8 (C-4′), 126.1 (C-3′, -5′), 111.7 (C-4a), 79.4 (C-2), 61.9 (OCH_3_-7), 61.7 (OCH_3_-8), 43.6 (C-3) (Figure S5).

Onysilin (**6**): ^1^H NMR (500 MHz, CDCl_3_); δ 11.88 (1H, s, OH-5), 7.38–7.47 (5H, m, H-2′-6′), 6.13 (1H, s, H-8), 5.42 (1H, dd, *J* = 13.2, 3.0 Hz, H-2), 3.88 (3H, s, OCH_3_-7), 3.85 (3H, s, OCH_3_-6), 3.10 (1H, dd, *J* = 17.3, 13.3 Hz, H-3α), 2.83 (1H, dd, *J* = 17.3, 3.0 Hz, H-3β). ^13^C NMR (125 MHz, CDCl_3_); δ 196.5 (C-4), 161.2 (C-7), 158.9 (C-8a), 155.2 (C-5), 138.5 (C-1′), 130.8 (C-6), 129.2 (C-4′), 128.9 (C-3′, -5′), 126.3 (C-2′, -6′), 103.4 (C-4a), 91.9 (C-8), 79.8 (C-2), 61.1 (OCH_3_-6), 56.4 (OCH_3_-7), 43.6 (C-3) (Figure S5).

Dihydropashanone (**7**): ^1^H NMR (500 MHz, CDCl_3_); δ 13.44 (1H, s, OH-2′), 7.19–7.31 (5H, m, H-2-6), 6.78 (1H, s, OH-6′), 6.06 (1H, s, H-3′), 3.90 (3H, s, OCH_3_-4′), 3.84 (3H, s, OCH_3_-5′) 3.43 (2H, t, *J* = 7.6 Hz, H-α), 3.04 (2H, t, *J* = 7.6 Hz, H-β). ^13^C NMR (125 MHz, CDCl_3_); δ 204.5 (C-β′), 162.0 (C-4′), 158.1 (C-6′), 151.2 (C-2′), 141.6 (C-5′), 128.5 (C-3, -5), 128.4 (C-2, -6), 127.8 (C-1), 126.0 (C-4), 103.7 (C-1′), 92.7 (C-3′), 61.4 (OCH_3_-4′), 55.9 (OCH_3_-5′) 45.2 (C-α), 30.4 (C-β) (Figure S6).

2′-Hydroxy-3′,4′,6′-trimethoxydihydrochalcone (**8**): ^1^H NMR (500 MHz, CDCl_3_); δ 13.92 (1H, s, OH-6′), 7.20–7.33 (5H, m, H-2-6), 5.98 (1H, s, H-3′), 3.95 (3H, s, OCH_3_-2′), 3.89 (3H, s, OCH_3_-4′), 3.83 (3H, s, OCH_3_-5′) 3.34 (2H, t, *J* = 7.7 Hz, H-α), 3.01 (2H, t, *J* = 7.7 Hz, H-β). ^13^C NMR (125 MHz, CDCl_3_); δ 205.3 (C-β′), 159.1 (C-2′), 159.0 (C-4′), 158.5 (C-6′), 141.9 (C-1), 130.9 (C-3′), 128.67 (C-2, -6), 128.66 (C-3, -5), 126.2 (C-4), 106.3 (C-1′), 86.6 (C-5′), 61.0 (OCH_3_-5′), 56.2 (OCH_3_-2′), 55.8 (OCH_3_-4′), 46.2 (C-α), 30.8 (C-β) (Figure S7).

Avicularin (**9**): HRESIMS; 301.0833 [M-ribose-H]^−^, 433.1268 [M-H]^−^, 867.2936 [2M-H]^−^, 1301.5210 [3M-H]^−^ (Figure S8).

Afzelin (**10**): HRESIMS; 285.0912 [M-rhamnose-H]^−^, 431.1403 [M-H]^−^, 863.3538 [2M-H]^−^, 1295.6177 [3M+H]^−^ (Figure S9).

Avicularin-acetate (**11**): HRESIMS; 300.0726 [M-acetylribose-H]^−^, 475.1339 [M − H]^−^, 951.3165 [2M+H]^−^, 1427.5588 [3M+H]^−^ (Figures S10 and S11).

Quercetrin (**12**): HRESIMS; 301.0961 [M-rhamnose-H]^−^, 447.1446 [M+H]^−^, 895.3333 [2M+H]^−^, 1343.5986 [3M+H]^−^ (Figure S12).

Linderone (**13**): ^1^H NMR (500 MHz, CDCl_3_); δ 11.56 (1H, s, OH-6), 7.67 (2H, s, H-7, -8) 7.62 (2H, dd, *J* = 8.0, 2.0 Hz, H-2′, -6′), 7.40 (3H, m, H-3′, -4′, -5′), 4.22 (3H, s, OCH_3_-4), 4.17 (3H, s, OCH_3_-5). ^13^C NMR (125 MHz, CDCl_3_); δ 193.5 (C-1), 185.0 (C-3), 164.8 (C-6), 148.5 (C-4), 145.6 (C-5), 141.4 (C-8), 135.2 (C-1′), 129.6 (C-2′, -6′), 129.0 (C-4′), 128.7 (C-3′, -5′), 117.7 (C-7), 102.0 (C-2), 60.0 (OCH_3_-4), 59.8 (OCH_3_-5) (Figure S13).

Methyllinderone (**14**): ^1^H NMR (500 MHz, CDCl_3_); δ 7.95 (1H, d, *J* = 15.8 Hz, H-8), 7.61 (2H, d, *J* = 6.6 Hz, H-2′, -6′), 7.53 (1H, d, *J* = 15.8 Hz, H-7), 7.38 (3H, m, H-3′, -4′, -5′), 4.20 (6H, s, OCH_3_-4, -5), 4.11 (3H, s, OCH_3_-6). ^13^C NMR (125 MHz, CDCl_3_); δ 187.4 (C-1), 184.9 (C-3), 165.6 (C-6), 149.1 (C-5), 148.0 (C-4), 141.5 (C-8), 135.8 (C-1′), 130.2 (C-4′), 129.1 (C-2′, -6′), 128.5 (C-3′, -5′), 121.4 (C-7), 109.6 (C-2), 64.5 (OCH_3_-6), 60.2 (OCH_3_-5), 60.1 (OCH_3_-4) (Figure S14).

Methyllucidone (**15**): ^1^H NMR (500 MHz, CDCl_3_); trans-: δ 8.01 (1H, d, *J* = 15.8 Hz, H-8), 7.61 (1H, m, H-7), 7.36–7.43 (5H, m, H-2′-6′), 5.95 (1H, s, H-5), 4.21 (3H, s, OCH_3_-4), 3.94 (3H, s, OCH_3_-6); cis-: δ 7.93 (1H, d, *J* = 15.8 Hz, H-8), 7.61 (1H, m, H-7), 7.65–7.59 (5H, m, H-2′-6′), 5.94 (1H, s, H-5), 4.22 (3H, s, OCH_3_-4), 3.94 (3H, s, OCH_3_-6). ^13^C NMR (125 MHz, CDCl_3_); trans-: δ 191.89 (C-1), 185.64 (C-3), 170.22 (C-4), 169.03 (C-6), 142.91 (C-8), 135.61(C-1′), 130.56 (C-4′), 129.11 (C-2′, -6′), 128.77 (C-3′, -5′), 121.66 (C-7), 111.91 (C-5), 109.44 (C-2), 64.83 (OCH_3_-6), 58.76 (OCH_3_-4); cis-: δ 189.78 (C-1), 188.47 (C-3), 169.92 (C-4), 169.19 (C-6), 143.12 (C-8), 135.53(C-1′), 130.59 (C-4′), 129.15 (C-2′, -6′), 128.76 (C-3′, -5′), 121.22C-7), 112.39 (C-5), 109.69 (C-2), 65.17 (OCH_3_-6), 58.68 (OCH_3_-4) (Figure S15).

Linderaspirone A (**16**): ^1^H NMR (500 MHz, CDCl_3_); δ 7.31–7.22 (10H, m, H-10′-14′/10′-14′), 5.55 (2H, d, *J* = 10.3 Hz, H-2/2′), 5.44 (2H, d, *J* = 10.3 Hz, H-3/3′), 3.95/3.89 (12H, s, OCH_3_-6/6′, -7/7′), 3.51 (6H, s, OCH_3_-1/1′). ^13^C NMR (125 MHz, CDCl_3_); δ 194.9/192.4 (C-5/5′, -8/8′), 154.1 (C-1/1′), 153.4/152.1 (C-6/6′, -7/7′), 138.2 (C-9/9′), 129.8 (C-10/10′, -14/14′), 128.6 (C-11/11′, -13/13′), 127.9 (C-12/12′), 102.3 (C-2/2′), 66.8 (OCH_3_-1/1′), 59.72/59.70 (OCH_3_-6/6′, -7/7′), 55.6 (C-4/4′), 43.5 (C-3/3′) (Figure S16).

Bi-linderone (**17**): ^1^H NMR (500 MHz, CDCl_3_); δ 7.24–7.03 (10H, m, H-10-14/10′-14′), 6.15 (1H, ddd, *J* = 11.5, 2.5, 2.0 Hz, H-2′), 4.85 (1H, t, *J* = 2.0 Hz, H-2), 4.33 (1H, t, *J* = 2.0 Hz, H-3), 4.13/4.06 (6H, m, OCH_3_-16′/17′), 3.89 (1H, d, *J* = 11.5 Hz, H-3′), 3.76/3.62 (6H, s, OCH_3_-16/17), 3.60 (3H, s, OCH_3_-15). ^13^C NMR (125 MHz, CDCl_3_); δ 196.3/194.9 (C-5/8), 186.9/184.2 (C-5′/8′), 172.9 (C-1′), 154.9 (C-1), 153.7/153.0 (C-6/7), 147.9/147.0 (C-6′/7′), 140.3 (C-9), 136.4 (C-9′), 129.3–127.7 (C-10-14/10′-14′), 111.2 (C-4′), 96.6 (C-2), 65.2 (OCH_3_-15′), 60.2/59.8 (C-16′/17′), 59.7/59.5 (C-16/17), 59.1 (C-4), 55.1 (OCH_3_-15), 48.5 (C-3′), 46.1 (C-3), 43.4 (C-2′) (Figure S17).

### 4.3. Cell Culture

BV2 and RAW264.7 cells used in the experiment were donated by Professor Yun-Cheol Kim of Wonkwang University (Iksan, Korea). Cells were cultured in 100 mm dishes at a magnification of 5 × 10^6^ cells/dish. As the culture medium, α-minimum essential medium (BV2) and Roswell Park Memorial Institute-1640 (RAW264.7) containing 10% heat-inactivated fetal bovine serum and 1% penicillin G (100 U/mL) were used. Culture conditions were maintained at 37 °C and 5% CO_2_.

### 4.4. MTT Assay

The method to measure cell viability was performed as previously described using the MTT assay [53]. The absorbance of the 96-well plate containing dissolved formazan was measured at a wavelength of 540 nm using an enzyme-linked immunosorbent assay (ELISA) microplate reader (Molecular Devices, San Jose, CA, USA).

### 4.5. Measurement of NO Generation

The method of measuring the production of NO, an inflammatory substance, was performed using the Griess reaction described above [53]. A total of 18 compounds isolated from *L. erythrocarpa* were reacted by concentration, and the reaction solution was analyzed at a wavelength of 570 nm. 

### 4.6. PGE_2_ Assay

The PGE_2_ measurement method was performed as previously described using a kit commercially available from R&D Systems (Minneapolis, MN, USA) [53]. Compounds 16, 17 and 18 isolated from *L. erythrocarpa* were treated at a concentration of 40 μM, and the supernatant of each well in which an inflammatory reaction occurred was analyzed at a wavelength of 450 nm.

### 4.7. IL-6 and TNF-α Assay

Methods for measuring IL-6 and TNF-α were performed as previously described using kits commercially available from Bio-Legend (San Diego, CA, USA) [53]. Compounds 16, 17 and 18 isolated from *L. erythrocarpa* were treated at a concentration of 40 μM, and the supernatant of each well in which an inflammatory reaction occurred was analyzed at a wavelength of 450 nm.

### 4.8. Western Blot Analysis

The expression levels of the pro-inflammatory proteins iNOS and COX-2 were performed as described above [53]. Cell lysis was lysed with Tris-HCl buffer (20 mM, pH 7.4) containing the protease inhibitor mixture (5 mg/mL pepstatin A, 5 mg/mL aprotinin, 1 mg/mL chymostatin and 0.1 mM phenylmethanesulfonyl fluoride). The kit used for protein concentration determination was the Lowry Protein Assay Kit (Sigma-Aldrich, Saint Louis, MO, USA). Antibodies used were iNOS (Cayman Chemical, Ann Arbor, MI, USA) and Anti-COX2/Cyclooxygenase 2 (Abcam, Cambridge, UK).

### 4.9. Measurement of DNA-Binding Activity of NF-κB

NF-κB DNA binding activity was performed as previously described using nuclear extracts of cells [54]. Compounds 16, 17 and 18 isolated from *L. erythrocarpa* were treated at a concentration of 40 μM, and cytoplasmic and nuclear fractions were obtained from an inflammatory reaction occurred cell using a nuclear extraction kit (Cayman, Ann Arbor, MI, USA). They were then analyzed at a wavelength of 450 nm using the NF-κB transcription factor assay kit (Cayman, Ann Arbor, MI, USA). 

### 4.10. Statistical Analysis

All data were acquired from three independent experiments and expressed as mean ± standard deviation. Statistical analysis was performed using GraphPad Software Inc. (San Diego, CA, USA) and GraphPad Prism software, version 3.03. Mean difference was defined as one-way analysis of variance and Newman–Keuls post hoc test, and statistical significance was defined as *p* < 0.05.

## 5. Conclusions

In this study, chalcone-type compounds kanakugiol (**1**), 2′-hydroxy-3′,4′,6′-trimethoxychalcone (**2**), pashanone (**3**), dihydropashanone (**7**), 2′-hydroxy-3′,4′,6′-trimethoxydihydrochalcone (**8**), flavonol-type compounds kanakugin (**4**), 5-hydroxyl-7,8-dimethoxyflavanone (**5**), Onysilin (**6**), flavonol glycoside compounds avicularin (**9**), afzelin (**10**), avicularin-acetate (**11**), Quercetrin (**12**), cyclopentadione-type compounds linderone (**13**), methyllinderone (**14**), methyllucidone (**15**), dimer of linderone types linderaspirone A (**16**), bi-linderone (**17**), and demethoxy-bi-linderone (**18**) were isolated from *L. erythrocarpa* leaves. Among the isolated compounds, **1**–**4**, **7**, **8**, and **13**–**18** significantly inhibited nitrite production in LPS-induced BV2 microglia and RAW264.7. In particular, compounds **16**–**18** significantly inhibited the production of inflammatory mediators NO, PGE_2_, TNF-α, and IL-6, and the expression of iNOS and COX-2, which are pro-inflammatory proteins via reducing the NF-κB activity. Consequently, this research suggests that these compounds isolated from *L. erythrocarpa* leaves could be promising substances for the development of therapeutic agents for the treatment of neurodegenerative diseases in case of neuroinflammation.

## Figures and Tables

**Table 1:** NMR spectroscopic data of demethoxy-bi-linderone (**18**).

No.	Demethoxy-bi-Linderone (18)			
	δ_C_^a,b^		δ_H_^a,c^, Mult. (*J* in Hz)	HMBC
1	205			
2	43.8	α	3.86, m	1, 3, 4, 9, 2′
		β	2.66, dd (14.0, 4.0)	1, 4, 5, 8, 9, 10, 3′
3	47.2		3.65, dd (14.0, 4.0)	
4	58.8			
5, 8	196.1, 195.0			
6, 7	153.4, 153.1			
9	137.4			
10–14	128.7–128.2		7.03–7.24, m	3, 9
15, 16 (OMe)	59.61, 59.56		3.84, 3.62, s	6, 7
1’	169.3			
2’	54.5		6.53, d (13.0)	1, 4, 1′, 3′, 4′, 9′
3’	49.7		4.02, d (13.0)	1, 3, 4, 5, 8, 1′, 2′, 9′
4’	109.8			
5’, 8’	187.4, 183.6			
6’, 7’	148.3, 146.8			
9’	136.1			
10’–14’	128.7–128.2		7.03–7.24, m	
15’ (OMe)	65.8		3.89, s	1′
16’, 17’ (OMe)	60.1, 59.8		4.15, 4.08, s	6′, 7′

^a^ Recorded in CDCl_3_, ^b^ 100 MHz, ^c^ 400 MHz.

## Data Availability

The data presented in this study are available in this article. Other data supporting the findings of this study are available upon request from the corresponding author.

## Supplementary Materials

The following supporting information can be downloaded at: https://www.mdpi.com/article/10.3390/ijms23137122/s1.
