# Supplementary material for: Effects of Compounds Isolated from Lindera erythrocarpa on Anti-Inflammatory and Anti-Neuroinflammatory Action in BV2 Microglia and RAW264.7 Macrophage
Source: Int J Mol Sci. 2022 Jun 27;23(13):7122. doi: 10.3390/ijms23137122 (PMC9267112; doi:10.3390/ijms23137122)
Supplement: Supplementary file 1 [file ijms-23-07122-s001.zip › ijms-1753519-supplementary.pdf]

Supplementary Information

Effects of Compounds Isolated from *Lindera erythrocarpa* on Anti-Inflammatory and Anti-Neuroinflammatory Action in BV2 Microglia and RAW264.7 Macrophage

Chi-Su Yoon [1,2,†], Hwan Lee [3,†], Zhiming Liu [3], Hyeong-Kyu Lee [1] and Dong-Sung Lee [3,*]

[1] Natural Medicine Research Center, Korea Research Institute of Bioscience & Biotechnology (KRIBB), Cheongju-si, Chungcheongbuk-do 28116, Korea; ycs1991@naver.com (C.-S.Y.); brightjem6178@gmail.com (H.-K.L.)
[2] Department of Chemistry, University of Florida, Gainesville 32611, United States
[3] College of Pharmacy, Chosun University, Dong-gu, Gwangju 61452, Korea; ghksdldi123@hanmail.net (H.L.); lzmqust@126.com (Z.L.)
* Correspondence: dslee2771@chosun.ac.kr (D.-S.L.); Tel.: +82-63-230-6386 (D.-S.L.)
† These authors contributed equally to this work.

Contents

Figure S1. ^{1}H- and ^{13}C- NMR spectrum of compound **1**.

Figure S2. ^{1}H- and ^{13}C- NMR spectrum of compound **2**.

Figure S3. ^{1}H- and ^{13}C- NMR spectrum of compound **3**.

Figure S4. ^{1}H- and ^{13}C- NMR spectrum of compound **4**.

Figure S5. ^{1}H- and ^{13}C- NMR spectrum of compound **5** and **6**.

Figure S6. ^{1}H- and ^{13}C- NMR spectrum of compound **7**.

Figure S7. ^{1}H- and ^{13}C- NMR spectrum of compound **8**.

Figure S8. HR-ESI-MS spectrum of compound **9**.

Figure S9. HR-ESI-MS spectrum of compound **10**.

Figure S10. HR-ESI-MS spectrum of compound **11**.

Figure S11. ^{1}H- and ^{13}C- NMR spectrum of compound **11**.

Figure S12. HR-ESI-MS spectrum of compound **12**.

Figure S13. ^{1}H- and ^{13}C- NMR spectrum of compound **13**.

Figure S14. ^{1}H- and ^{13}C- NMR spectrum of compound **14**.

Figure S15. ^{1}H- and ^{13}C- NMR spectrum of compound **15**.

Figure S16. ^{1}H- and ^{13}C- NMR spectrum of compound **16**.

Figure S17. ^{1}H- and ^{13}C- NMR spectrum of compound **17**.

Figure S18. ^{1}H- and ^{13}C- NMR spectrum of compound **18**.

Figure S19. DEPT135 NMR spectrum of compound **18**.

Figure S20. HMQC spectrum of compound **18**.

Figure S21. COSY spectrum of compound **18**.

Figure S22. HMBC spectrum of compound **18**.

Figure S23. HR-ESI-MS spectrum of compound **18**.

Figure S24. The effects of compounds 16, 17, and 18 on the nitrite, PGE2, TNF-α and IL-6 production in BV2 (a, c, e, g) and RAW264.7 (b, d, f, h).

Table S1. NMR comparison with bi-linderone type of structures.

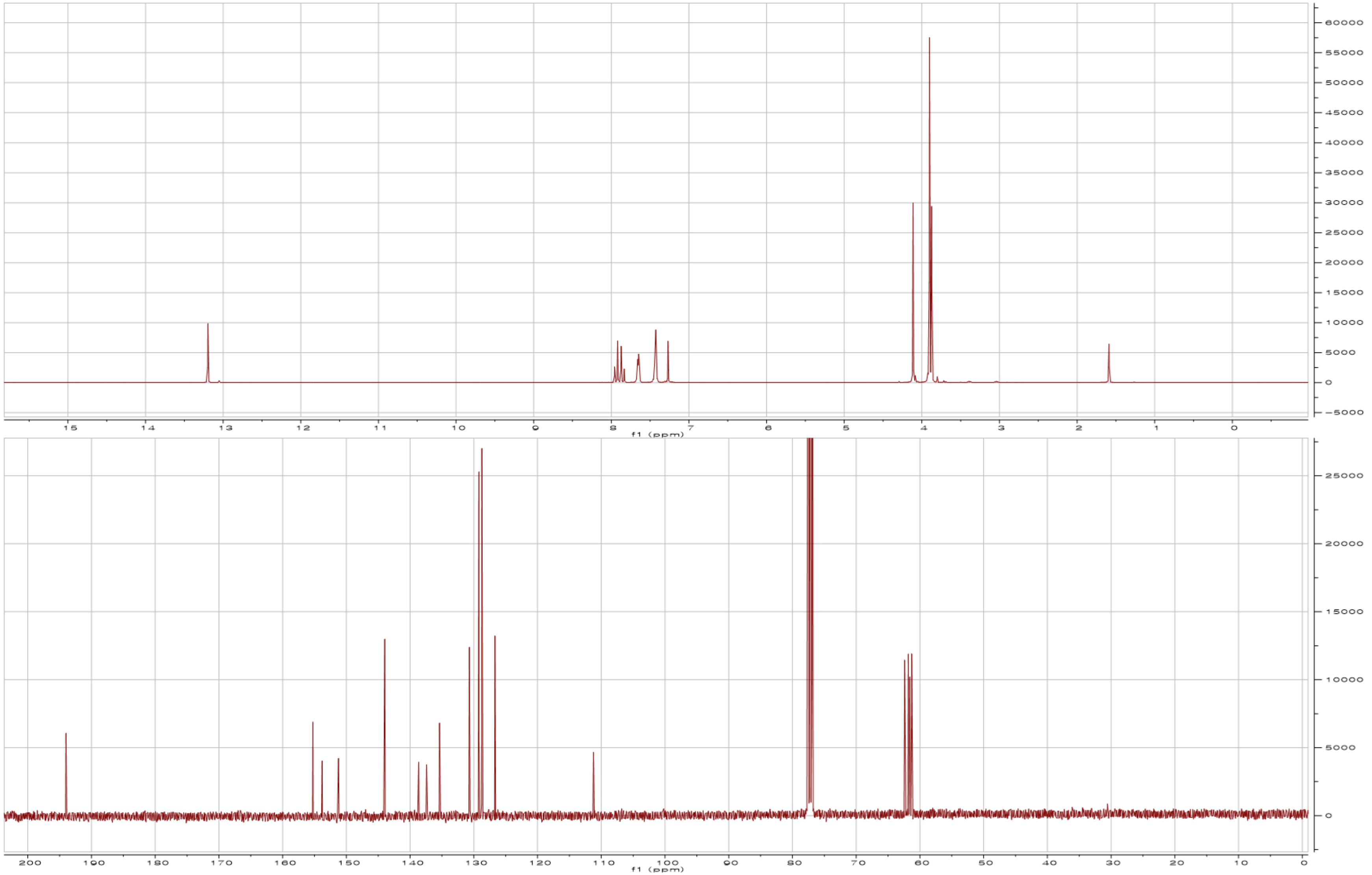

Figure S1. ^{1}H- and ^{13}C- NMR spectrum of compound **1**.

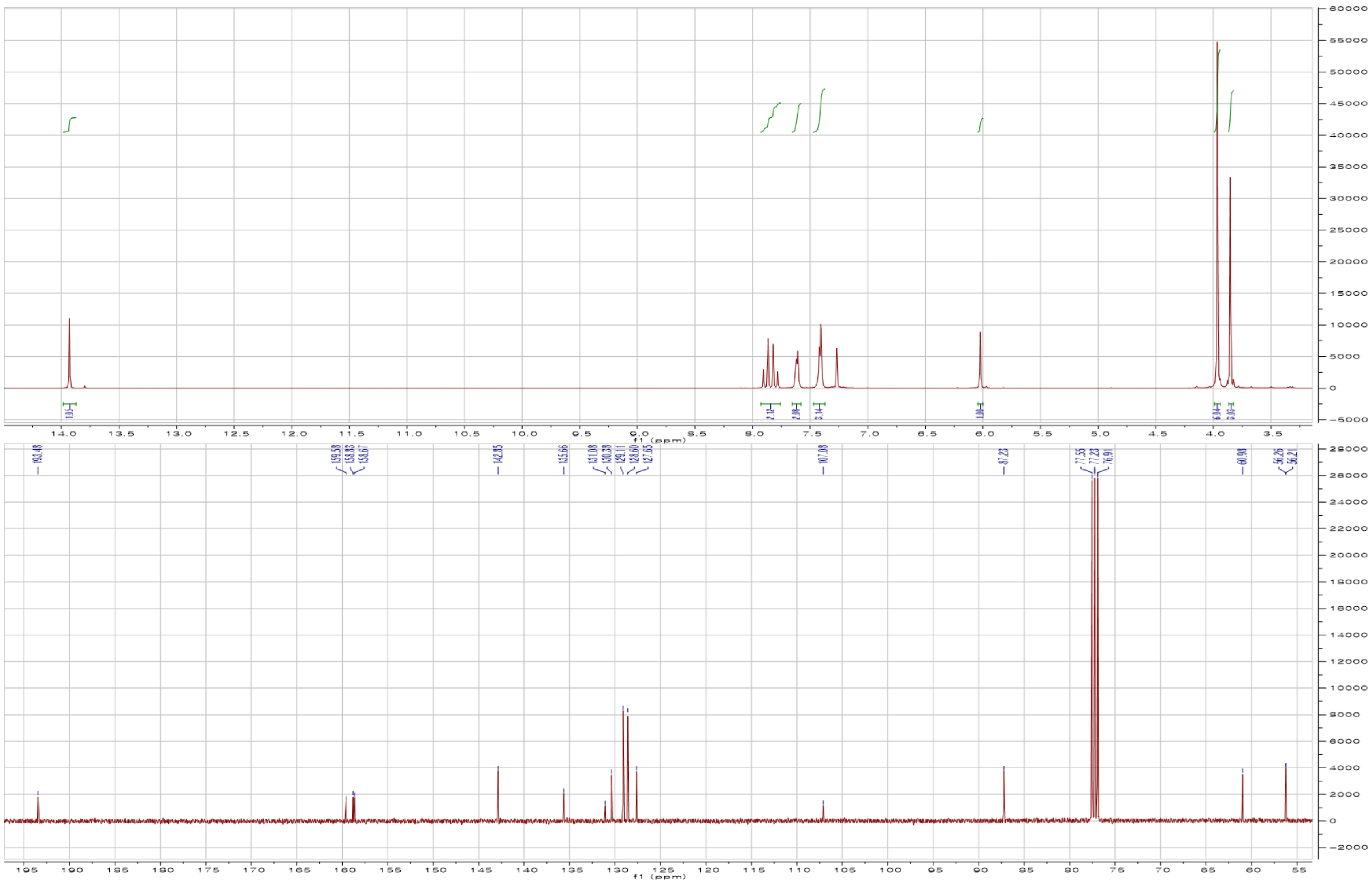

Figure S2. ^{1}H- and ^{13}C- NMR spectrum of compound **2**.

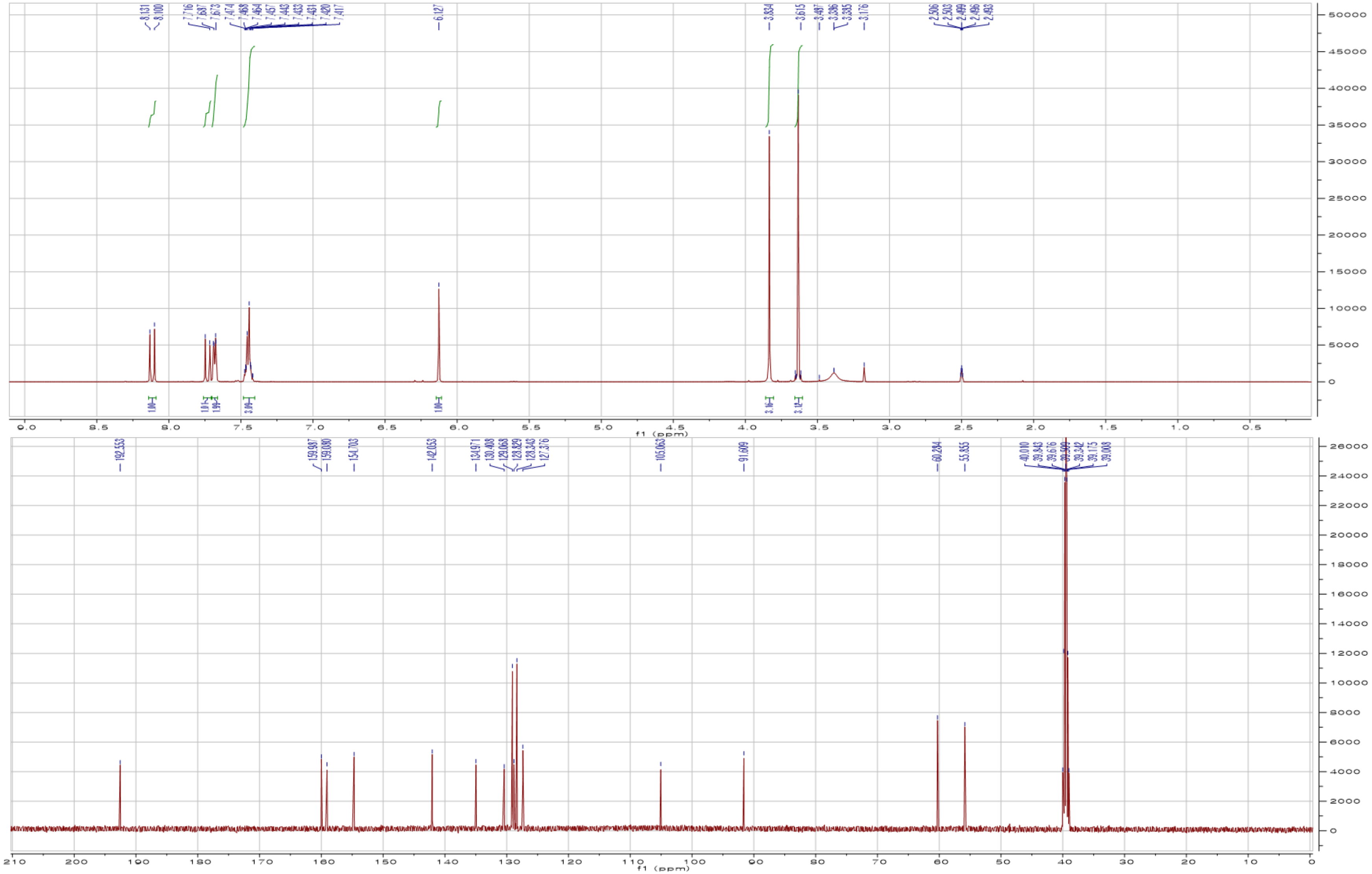

Figure S3. ^{1}H- and ^{13}C- NMR spectrum of compound **3**.

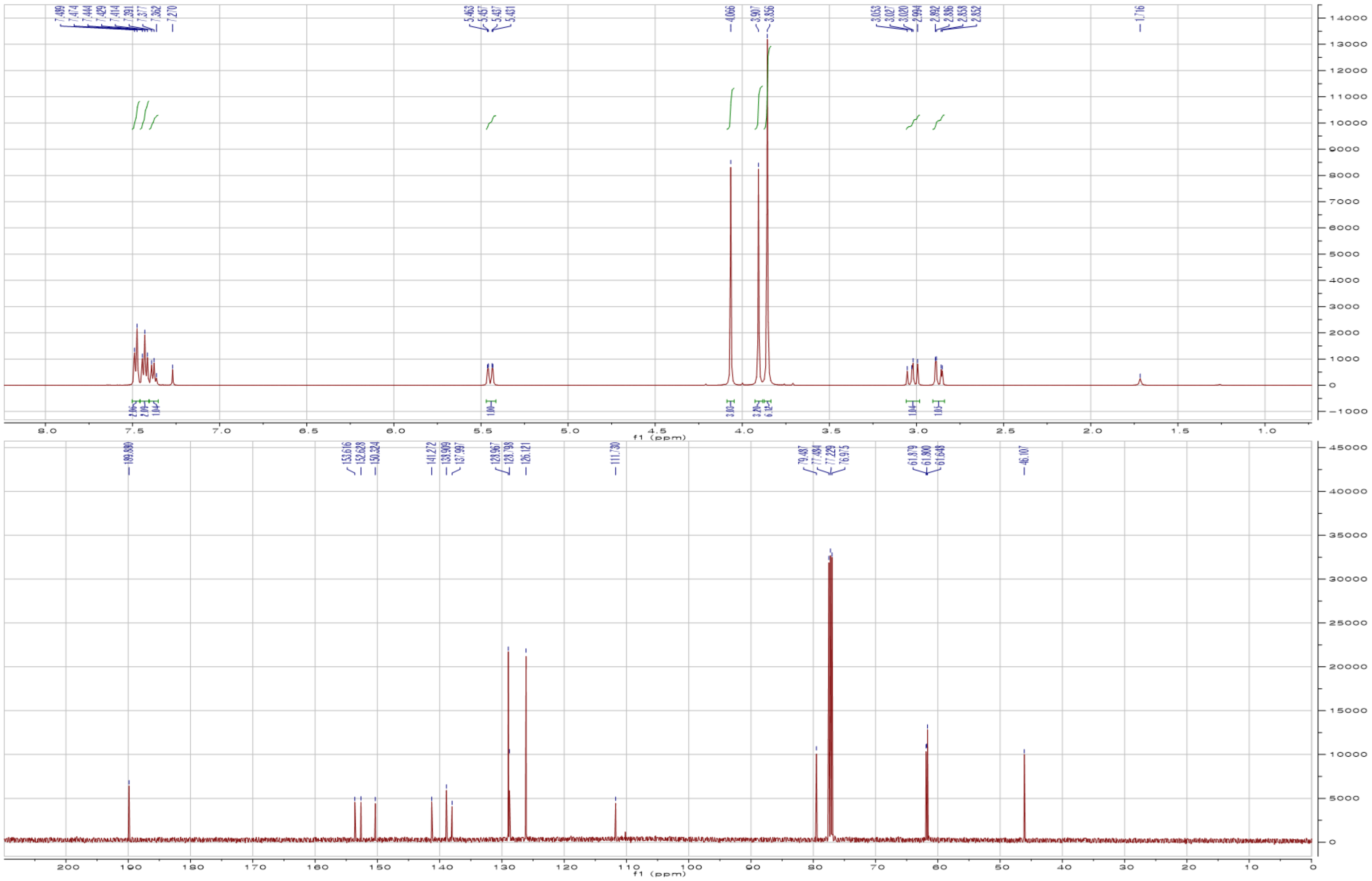

Figure S4. ^{1}H- and ^{13}C- NMR spectrum of compound **4**.

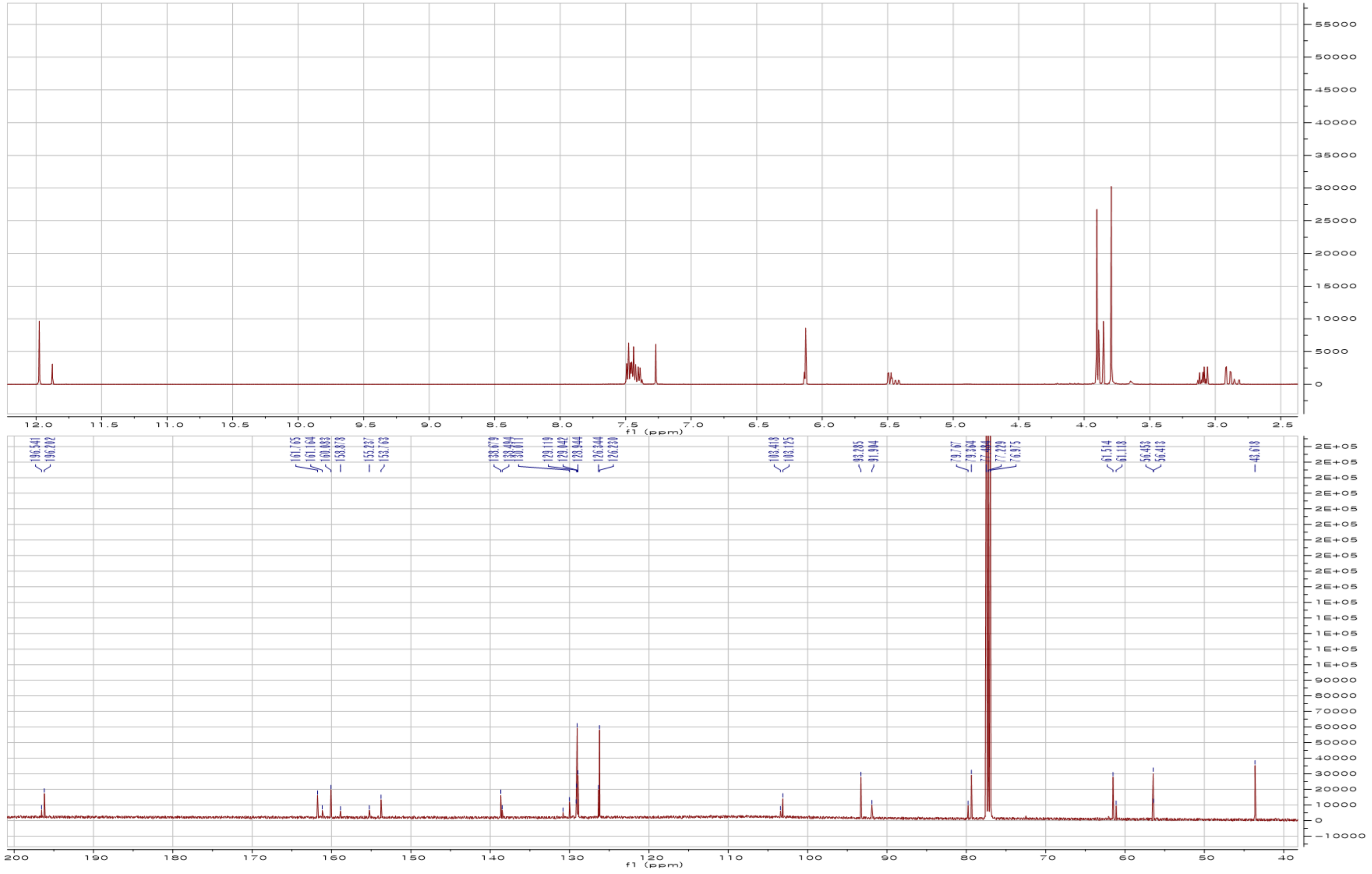

Figure S5. ^{1}H- and ^{13}C- NMR spectrum of compound **5** and **6**.

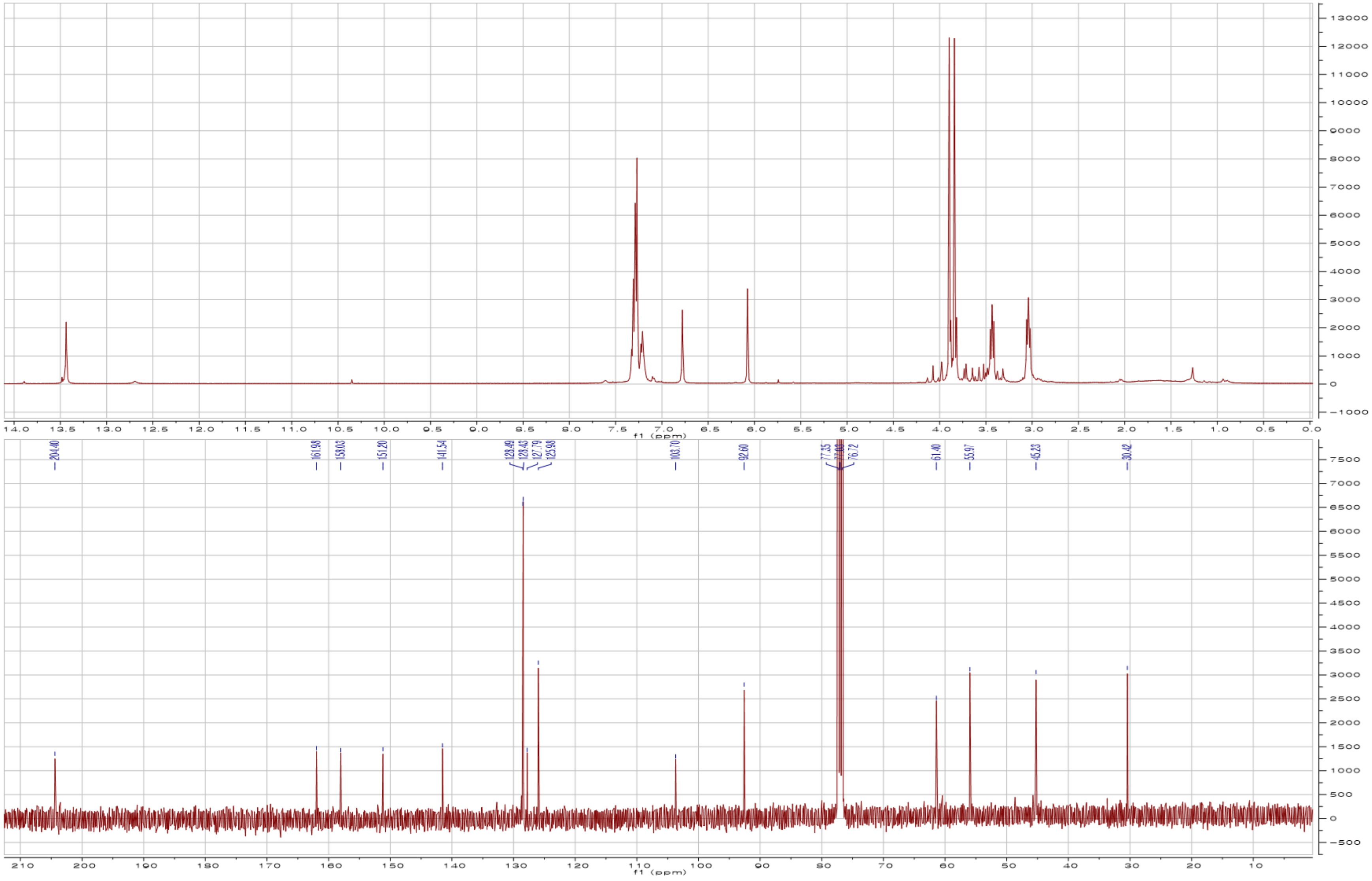

Figure S6. ^{1}H- and ^{13}C- NMR spectrum of compound **7**.

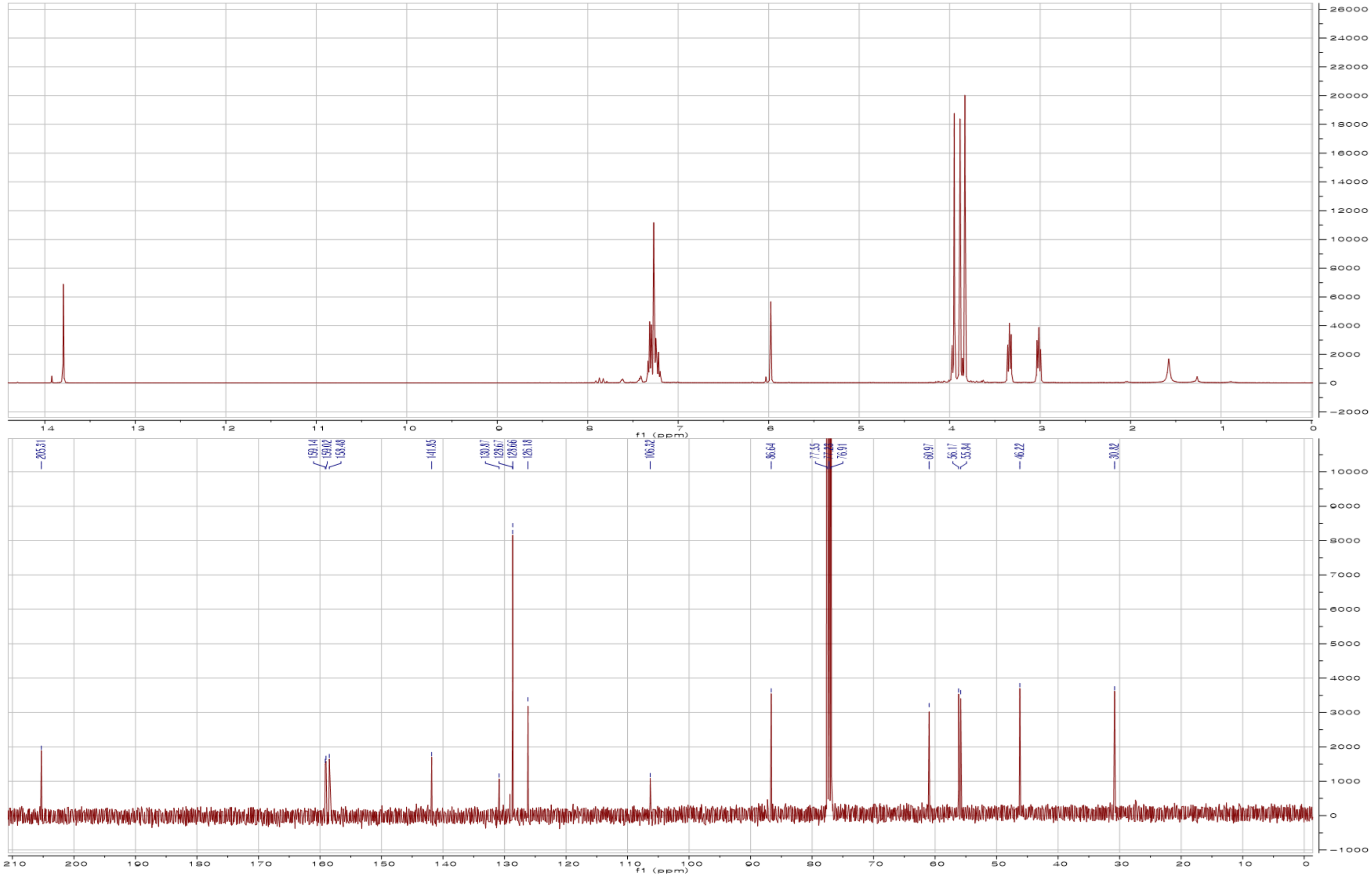

Figure S7. ^{1}H- and ^{13}C- NMR spectrum of compound **8**.

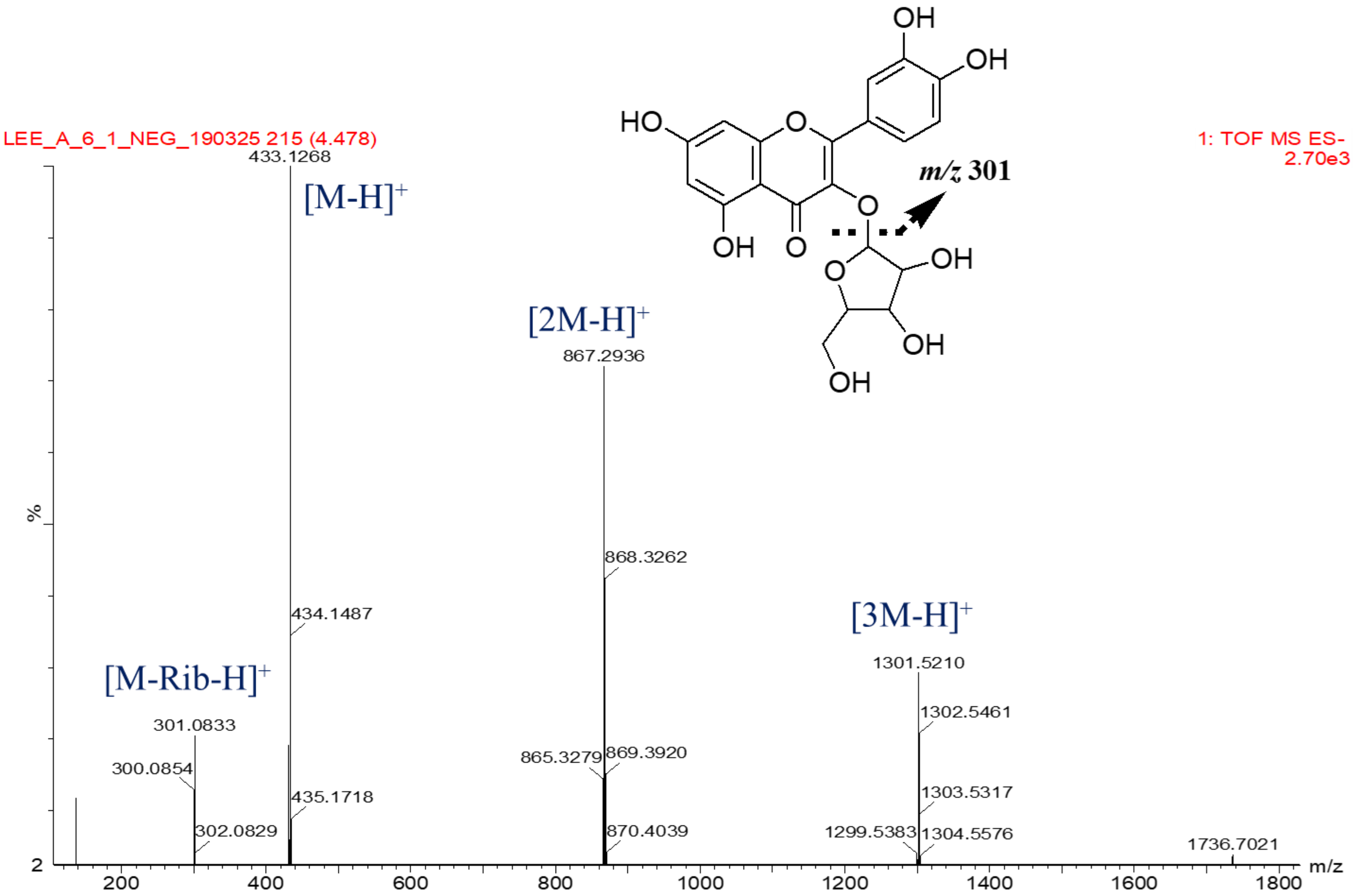

Figure S8. HR-ESI-MS spectrum of compound **9**.

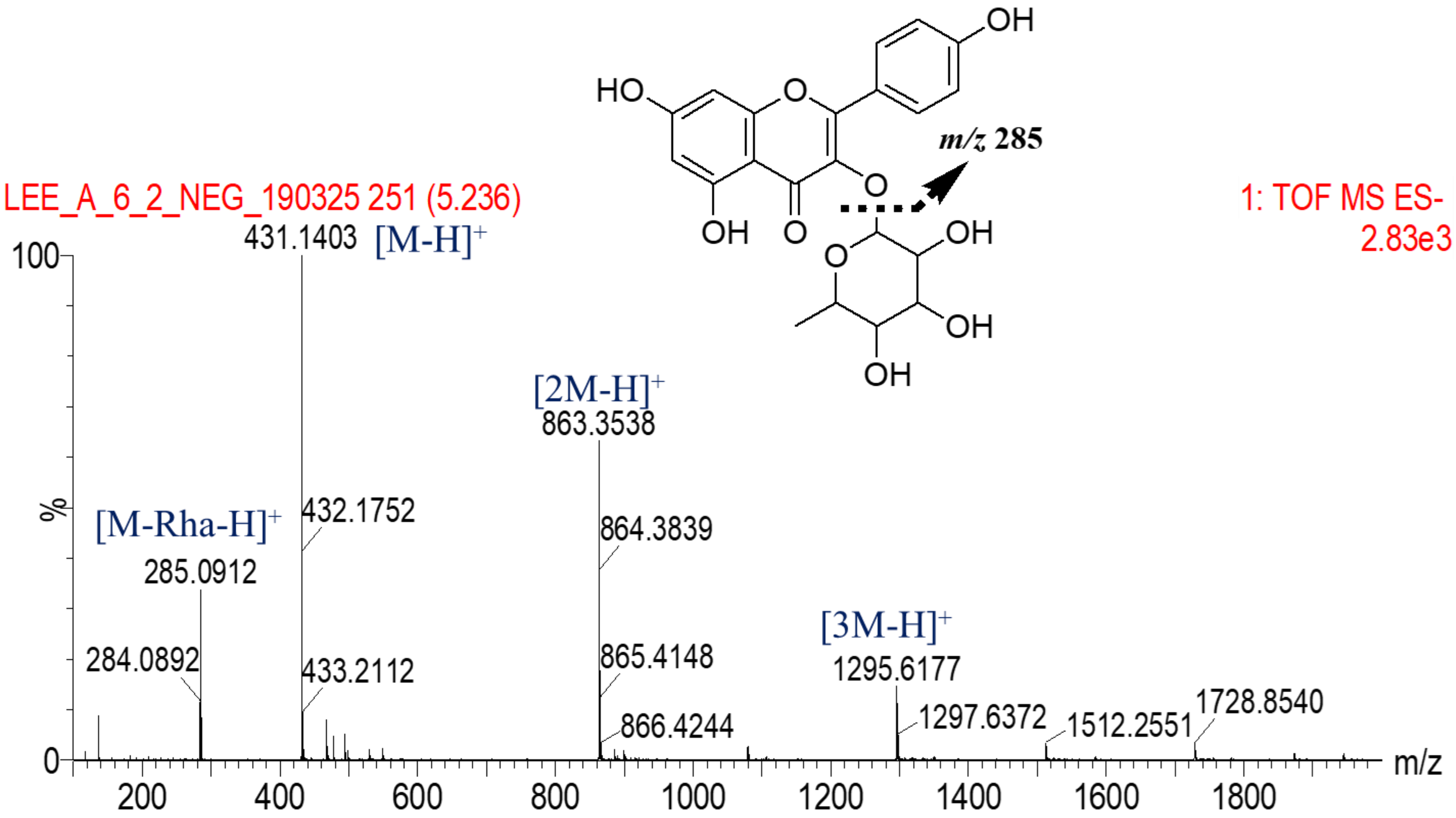

Figure S9. HR-ESI-MS spectrum of compound **10**.

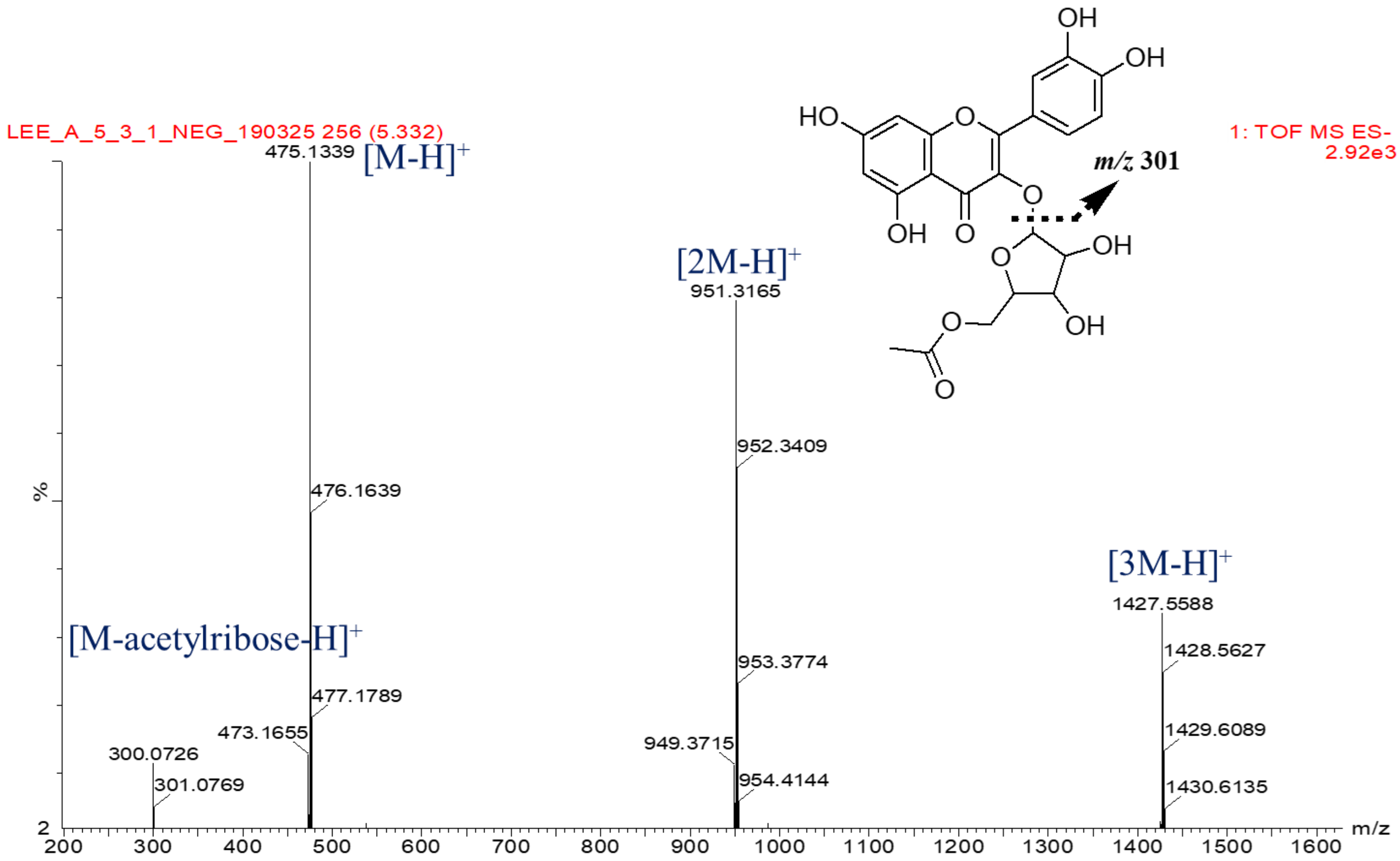

Figure S10. HR-ESI-MS spectrum of compound **11**.

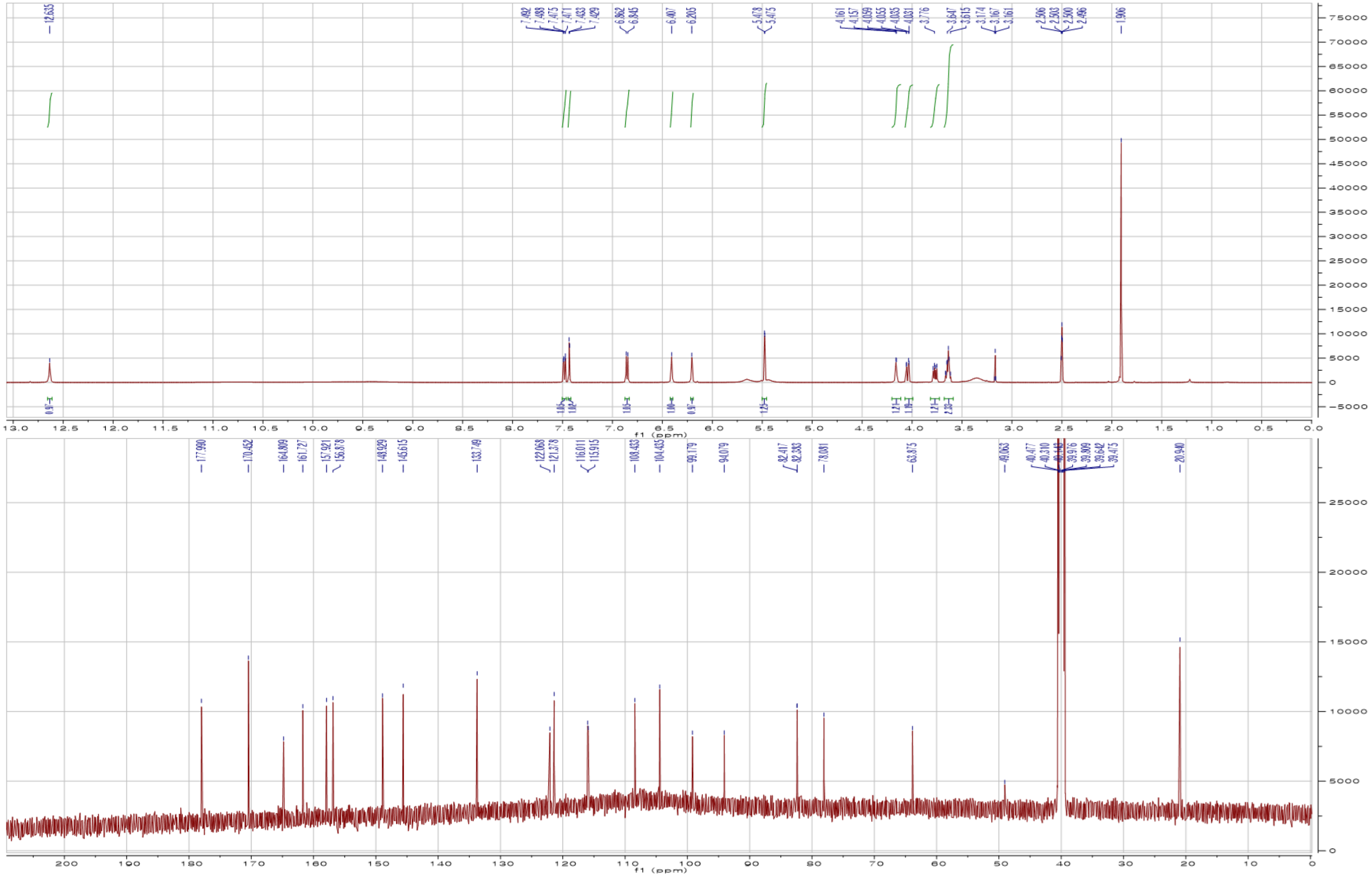

Figure S11. ^{1}H- and ^{13}C- NMR spectrum of compound **11**.

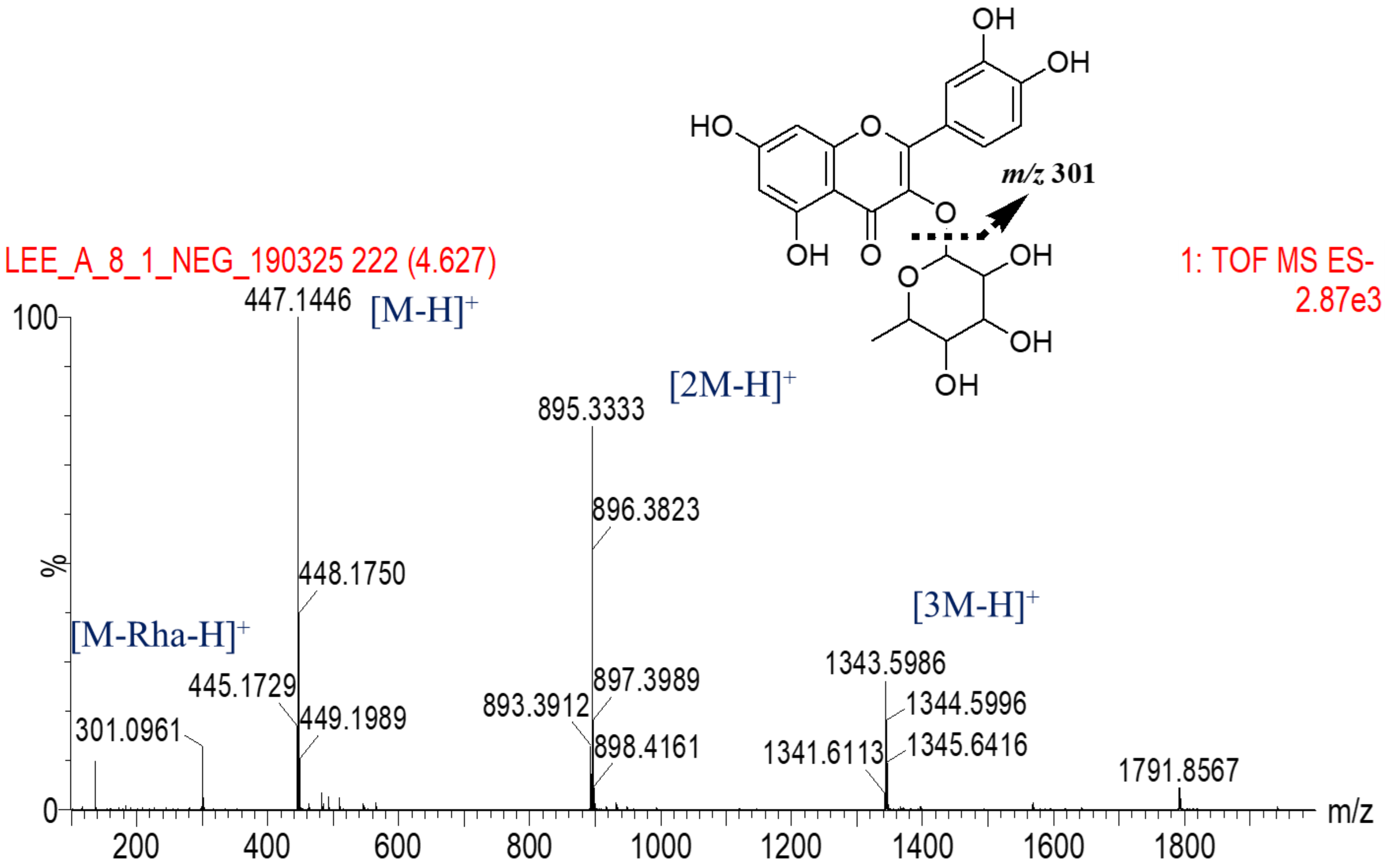

Figure S12. HR-ESI-MS spectrum of compound **12**.

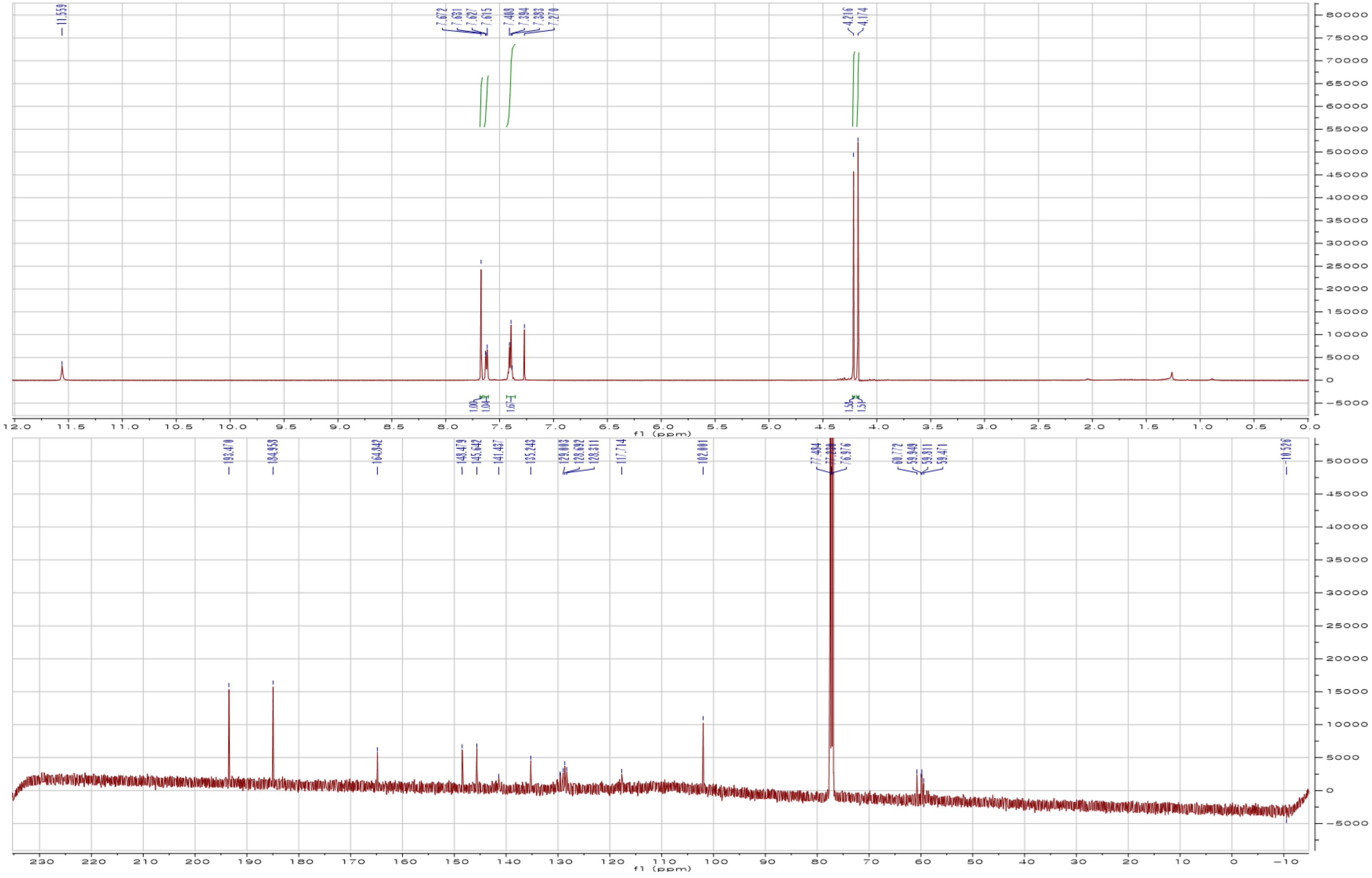

Figure S13. ^{1}H- and ^{13}C- NMR spectrum of compound **13**.

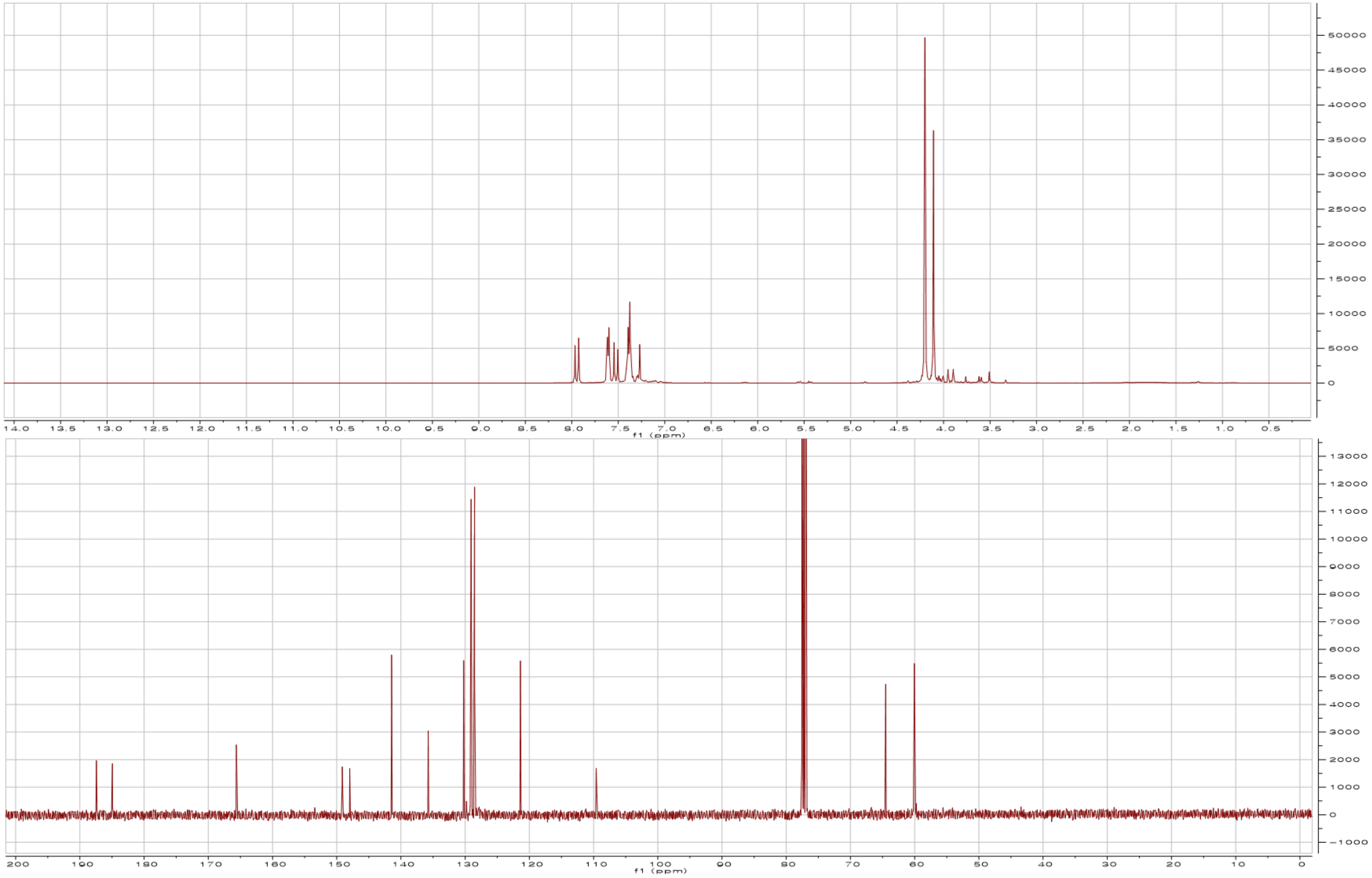

Figure S14. ^{1}H- and ^{13}C- NMR spectrum of compound **14**.

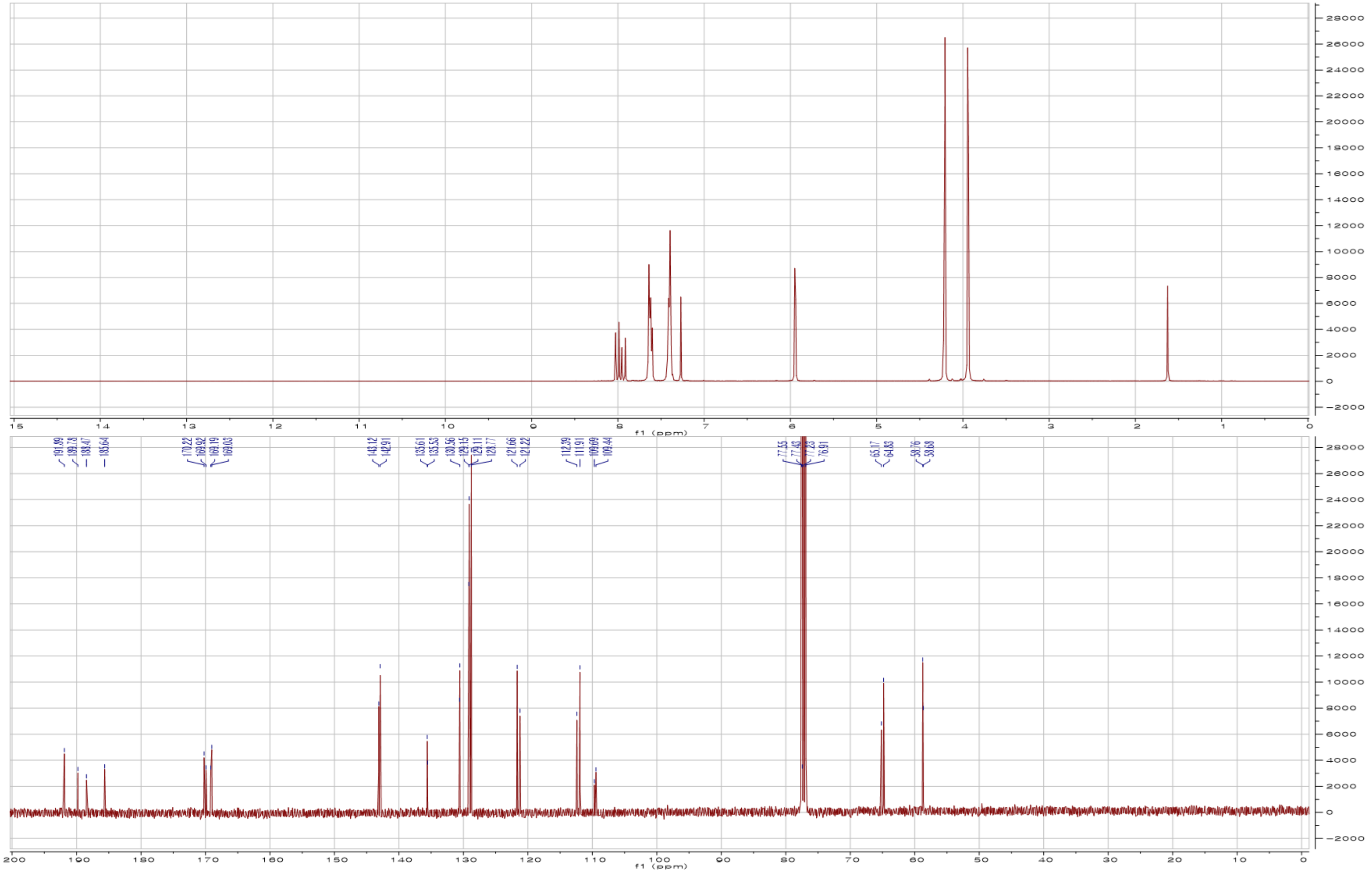

Figure S15. ¹H- and ¹³C- NMR spectrum of compound **15**.

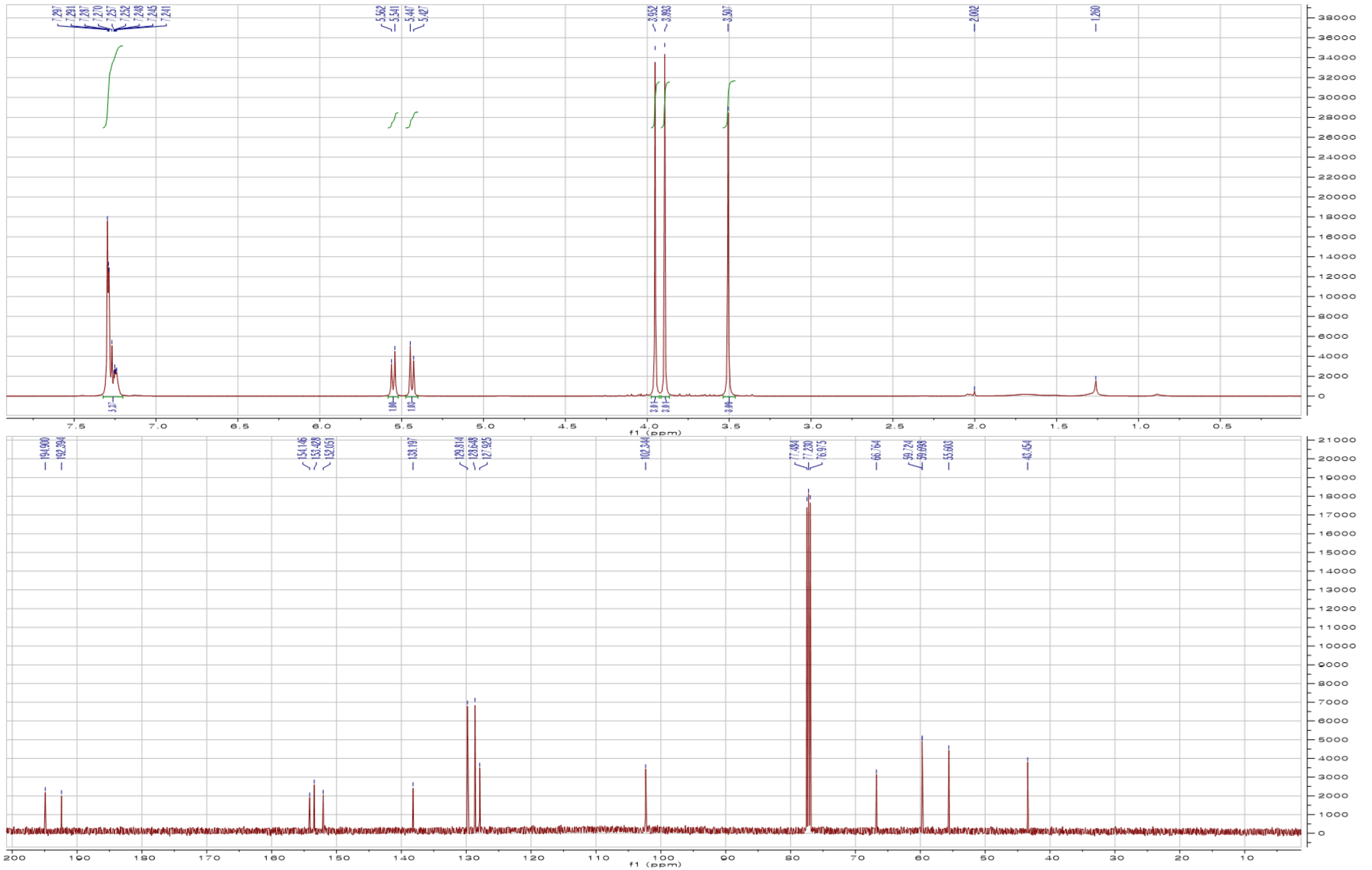

Figure S16. ^{1}H- and ^{13}C- NMR spectrum of compound **16**.

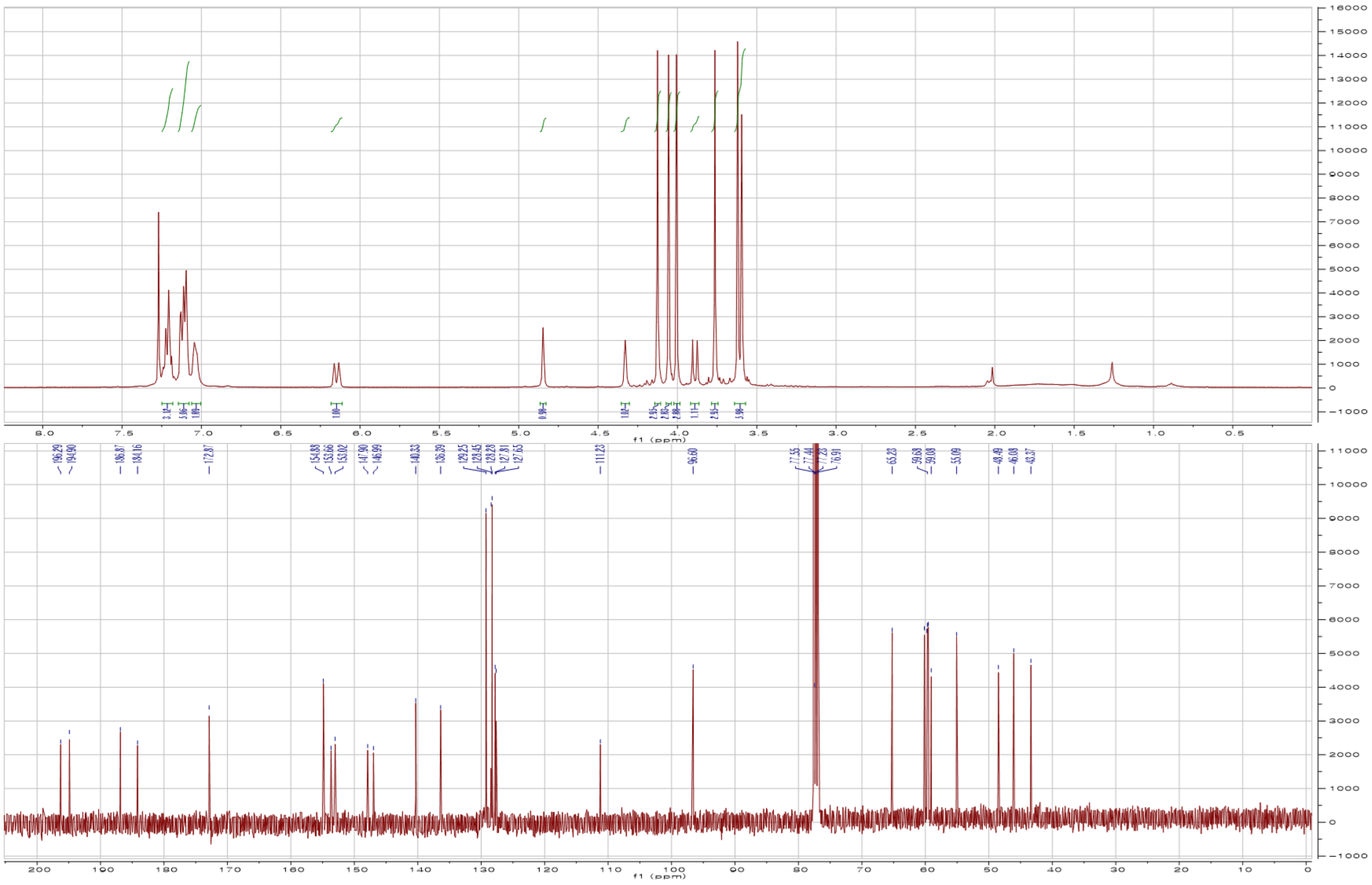

Figure S17. ^{1}H- and ^{13}C- NMR spectrum of compound **17**.

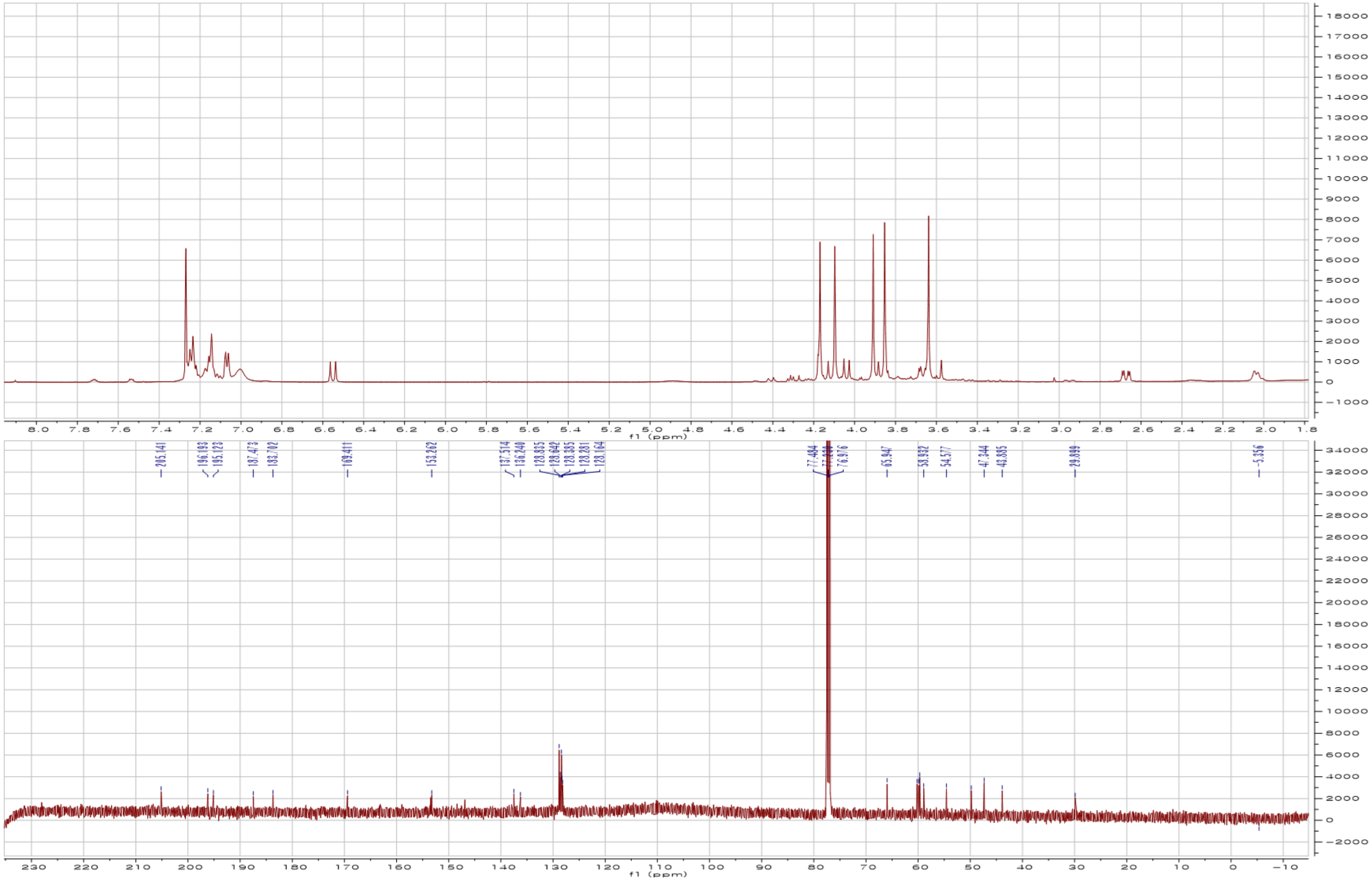

Figure S18. ^{1}H- and ^{13}C- NMR spectrum of compound **18**.

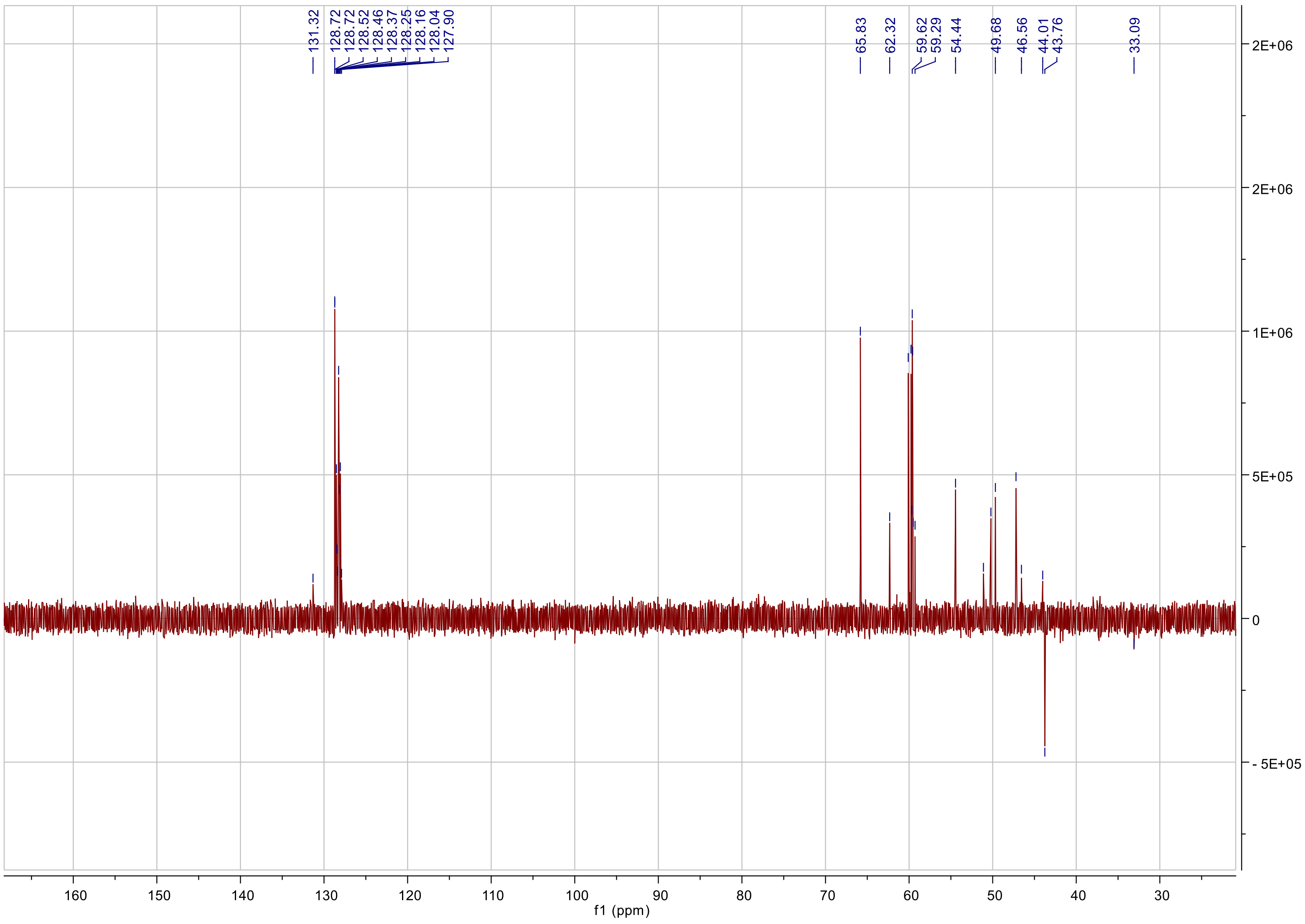

Figure S19. DEPT135 NMR spectrum of compound **18**.

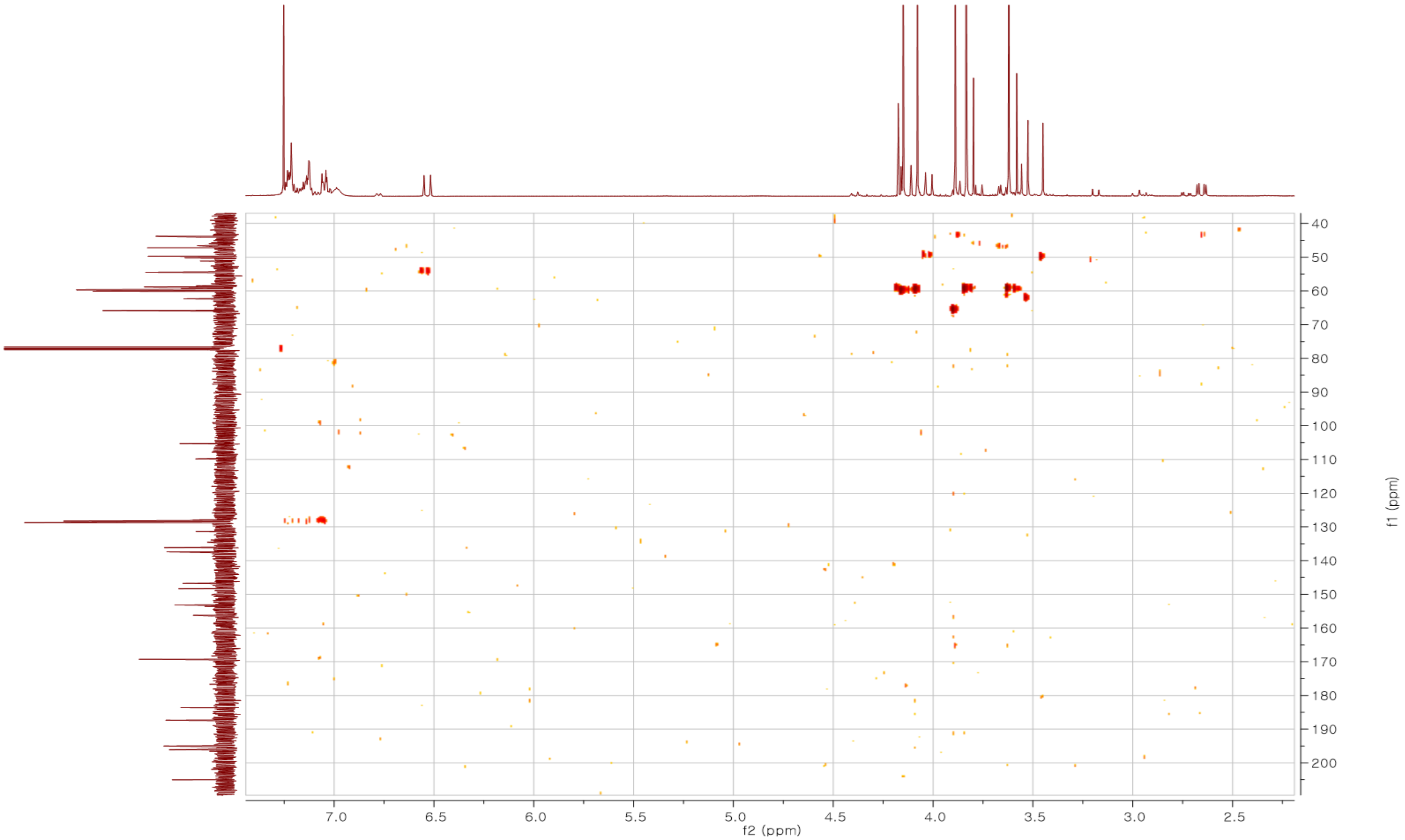

Figure S20. HMQC spectrum of compound **18**.

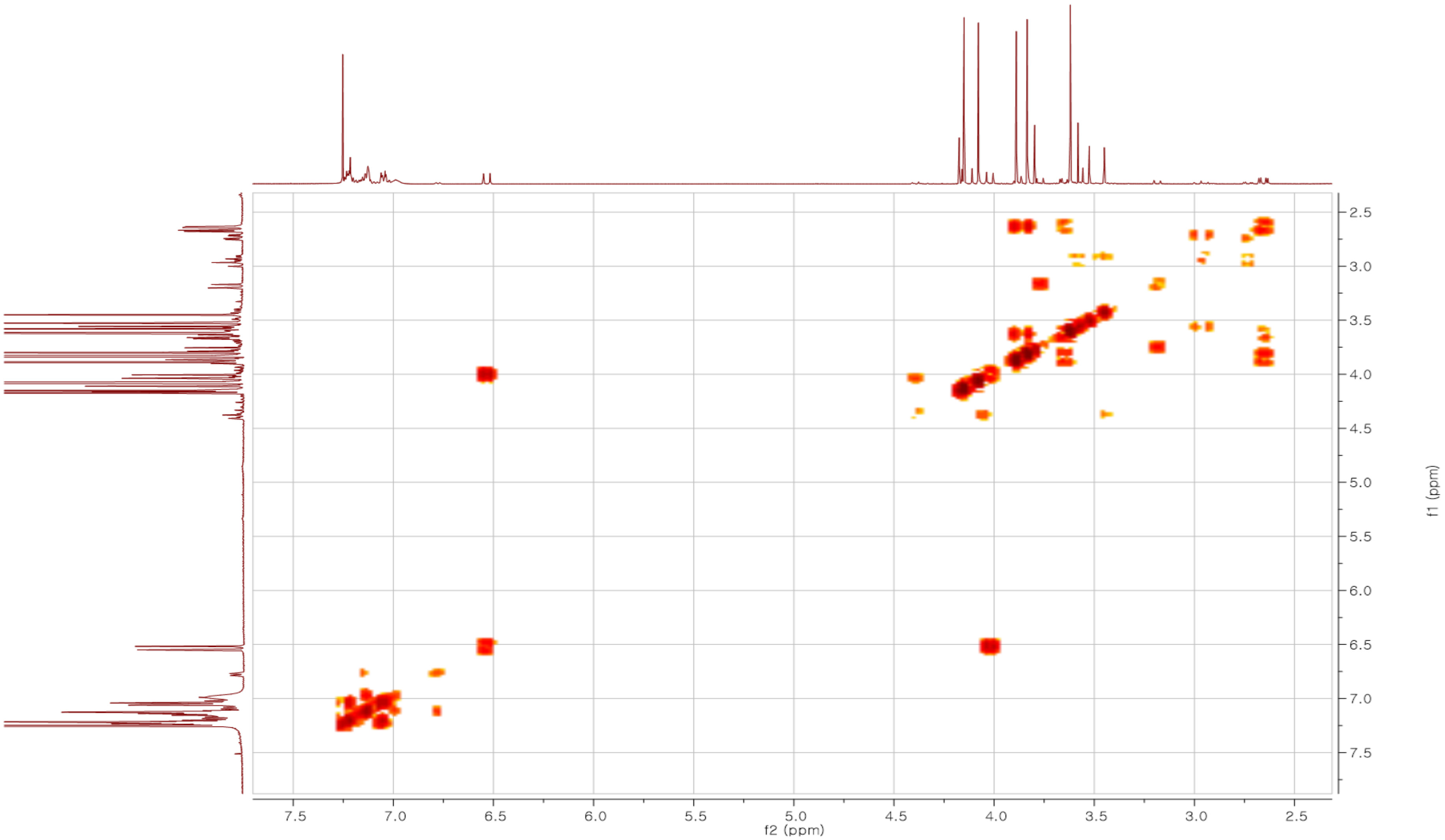

Figure S21. COSY spectrum of compound **18**.

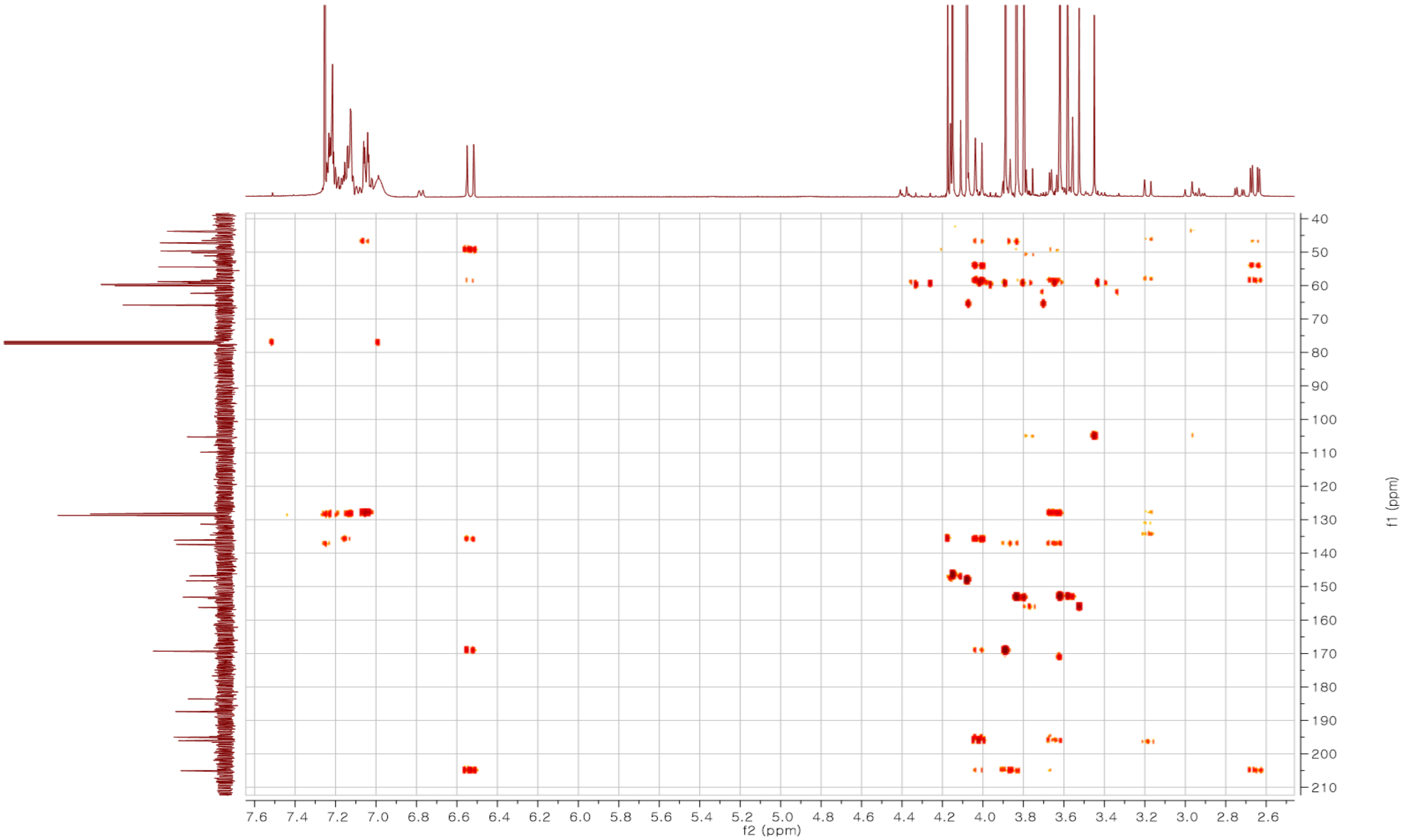

Figure S22. HMBC spectrum of compound **18**.

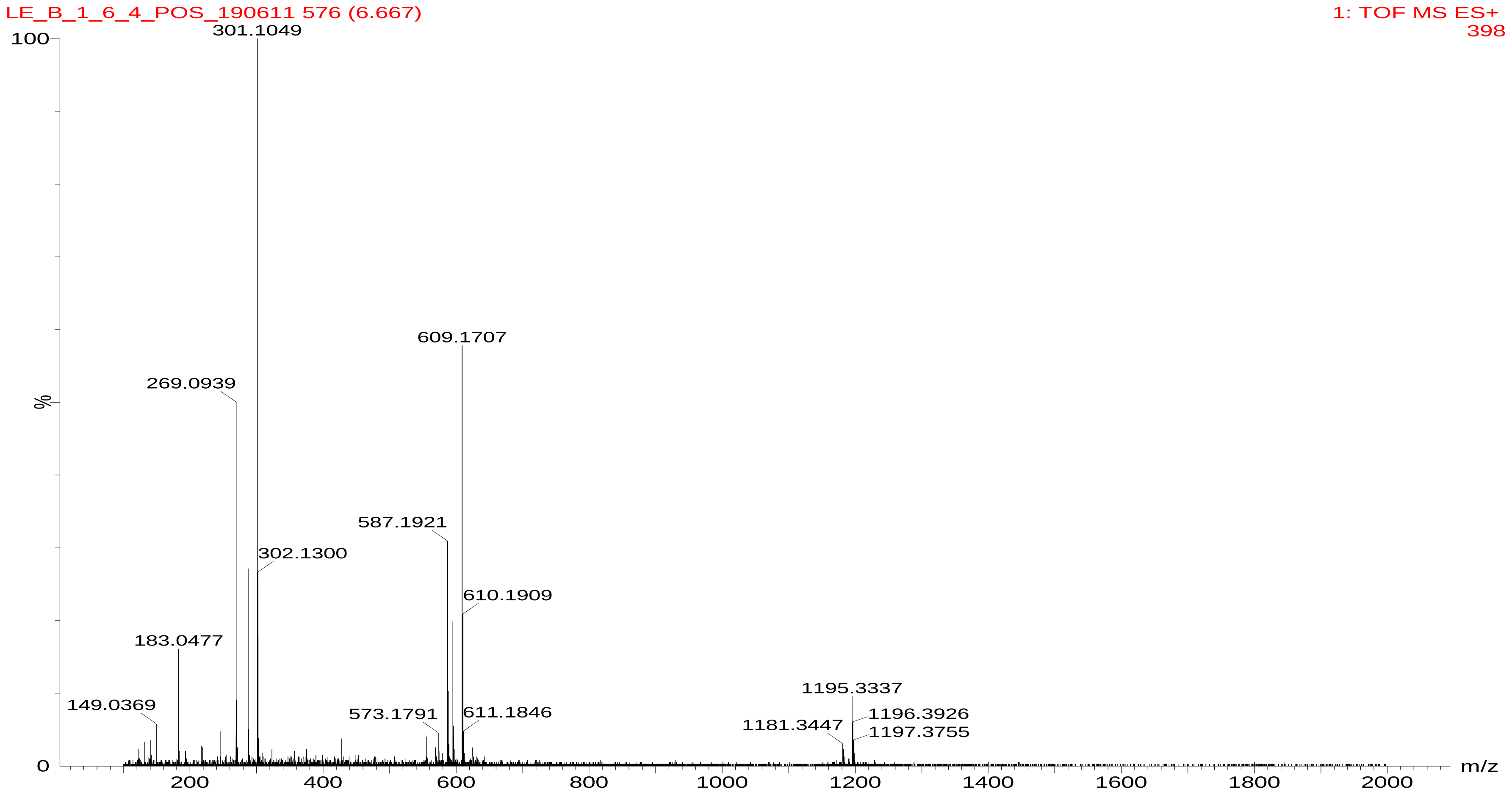

Figure S23. HR-ESI-MS spectrum of compound **18**.

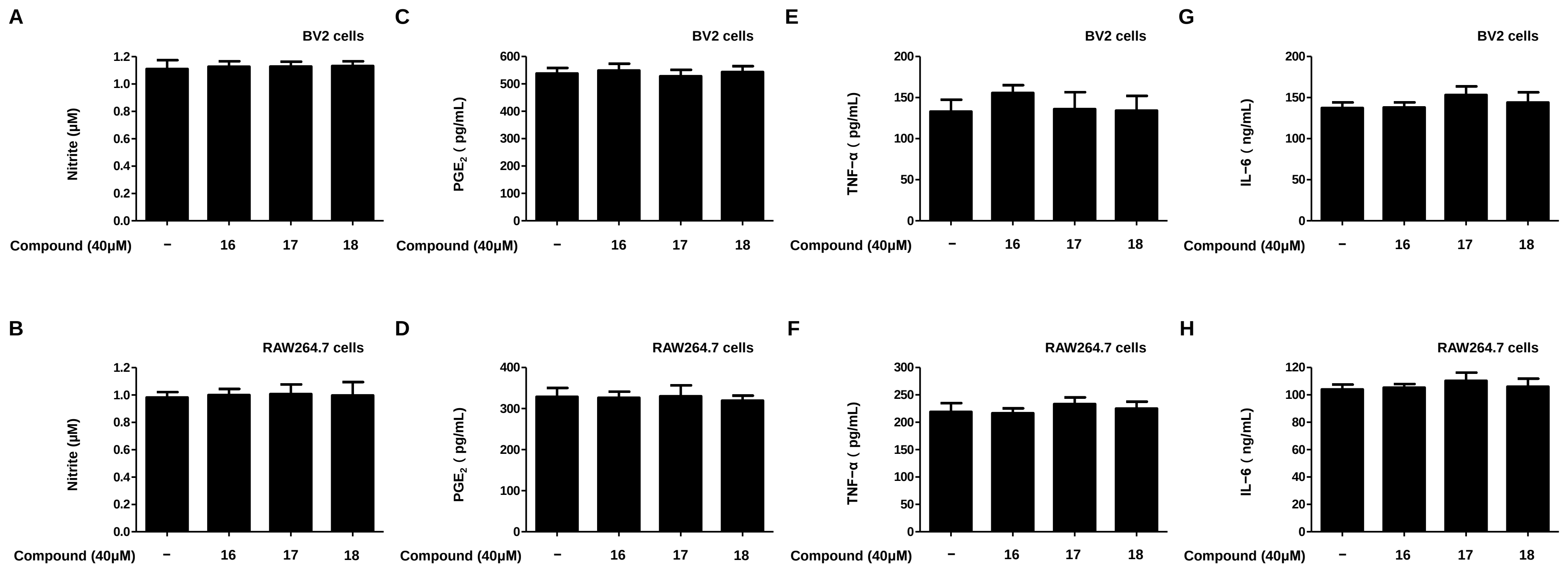

Figure S24. The effects of compounds 16, 17, and 18 on the nitrite, PGE2, TNF-*α* and IL-6 production in BV2 microglia (a, c, e, g) and RAW264.7 macrophages (b, d, f, h). In BV2 and RAW264.7 cells, the effect of Nitrite, PGE2, TNF-*α* and IL-6 production was induced for 18 h after pretreatment with 40 μM of the each compounds. Data are presented as mean ± standard deviation values of three independent experiments.

Table S1. NMR comparison with bi-linderone type of structures.

No	epi-bi-linderone[1]		bi-linderone[1]		demethoxy-epi-bi-linderone[2]			demethoxy-bi-linderone (**18**)	
	δ_C	δ_H, mult. (J= Hz)	δ_C	δ_H, mult. (J= Hz)	δ_C		δ_H, mult. (J= Hz)	δ_C	δ_H, mult. (J= Hz)
1	155.2		154.6		206.4			205.0	
2	95.6	4.84, br d (5.4)	96.3	4.84, br s	41.5	β<br>α	3.54, dd, (17.0, 14.0)<br>2.76, dd (17.0, 2.9)	43.8	3.86, m<br>2.66, dd (14.0, 4.0)
3	43.8	3.67, br d (5.4)	45.8	4.32, br s	41.2		3.97, dd (14.0, 2.9)	47.2	3.65, dd (14.0, 4.0)
4	56.9		58.8		58.7			58.8	
5, 8	195.5, 193.3		196.1, 194.7		195.9, 195.7			196.1, 195.0	
6, 7	151.6, 150.7		153.4, 152.8		153.2, 153.1			153.4, 153.1	
9	141.4		140.1		137.1			137.4	
10–14	127.2–129.6	6.90–7.45, m	127.4–129.0	7.00–7.24, m	127.9–128.8		7.02–7.24, m	128.7–128.2	7.03–7.24, m
15	54.8	3.58, s	54.9	3.58, s					
16, 17	59.5, 59.4	4.05, s, 3.73, s	59.3, 59.5	3.75, s, 3.61, s	59.8, 59.6		3.74, s, 3.66, s	59.61, 59.56	3.84, s, 3.62, s
1′	173.0		172.6		169.2			169.3	
2′	43.1	5.90, d (11.6)	43.1	6.14, br d (11.5)	52.8		6.39, d (13.3)	54.5	6.53, d (13.0)
3′	47.2	3.82, d (11.6)	48.2	3.88, d (11.5)	45.0		4.23, d (13.3)	49.7	4.02,d (13.0)
4′	111.1		111.0		110.7			109.8	
5′, 8′	186.3, 183.7		186.7, 183.9		187.5, 183.3			187.4, 183.6	
6′, 7′	147.8, 146.9		147.7, 146.7		148.7, 146.9			148.3, 146.8	
9′	136.5		136.1		135.7			136.1	
10′–14′	127.2–129.6	6.90–7.45, m	127.4–129.0	7.00–7.24, m	127.8–128.8		7.02–7.24, m	128.7–128.2	7.03–7.24, m
15′	65.7	4.09, s	65.0	3.99, s	65.8		3.65, s	65.8	3.89, s
16′, 17′	59.8, 59.5	4.15, s, 4.11, s	59.5, 59.9	4.11, s, 4.04, s	60.2, 59.7		4.20, s, 4.12, s	60.1, 59.8	4.15, s, 4.08, s

[1] *Org. Lett.* **2011**, *13*, 2192–2195. [2] *RSC Advances*. **2018**, *8*, 17898–17904.
